# Why we should care about movements: Using spatially explicit integrated population models to assess habitat source–sink dynamics

**DOI:** 10.1111/1365-2656.13357

**Published:** 2020-10-20

**Authors:** Matthieu Paquet, Debora Arlt, Jonas Knape, Matthew Low, Pär Forslund, Tomas Pärt

**Affiliations:** ^1^ Department of Ecology Swedish University of Agricultural Sciences Uppsala Sweden; ^2^ SLU Swedish Species Information Centre Swedish University of Agricultural Sciences Uppsala Sweden

**Keywords:** dispersal, habitat quality, integrated population model, land use, *Oenanthe oenanthe*, population dynamics, sex differences, source–sink

## Abstract

1. Assessing the source–sink status of populations and habitats is of major importance for understanding population dynamics and for the management of natural populations. Sources produce a net surplus of individuals (per capita contribution to the metapopulation > 1) and will be the main contributors for self‐sustaining populations, whereas sinks produce a deficit (contribution < 1). However, making these types of assessments is generally hindered by the problem of separating mortality from permanent emigration, especially when survival probabilities as well as moved distances are habitat‐specific.
2. To address this long‐standing issue, we propose a spatial multi‐event integrated population model (IPM) that incorporates habitat‐specific dispersal distances of individuals. Using information about local movements, this IPM adjusts survival estimates for emigration outside the study area.
3. Analysing 24 years of data on a farmland passerine (the northern wheatear *Oenanthe oenanthe*), we assessed habitat‐specific contributions, and hence the source–sink status and temporal variation of two key breeding habitats, while accounting for habitat‐ and sex‐specific local dispersal distances of juveniles and adults. We then examined the sensitivity of the source–sink analysis by comparing results with and without accounting for these local movements.
4. Estimates of first‐year survival, and consequently habitat‐specific contributions, were higher when local movement data were included. The consequences from including movement data were sex specific, with contribution shifting from sink to likely source in one habitat for males, and previously noted habitat differences for females disappearing.
5. Assessing the source–sink status of habitats is extremely challenging. We show that our spatial IPM accounting for local movements can reduce biases in estimates of the contribution by different habitats, and thus reduce the overestimation of the occurrence of sink habitats. This approach allows combining all available data on demographic rates and movements, which will allow better assessment of source–sink dynamics and better informed conservation interventions.

## Graphical abstract

To access the source‐sink status of populations and habitats, the authors developed a novel integrated population model (IPM) that adjusts survival estimates for emigration outside the study area by using information about local movements. The authors show that spatial‐IPM can allow better assessment of source‐sink dynamics and better informed conservation interventions.

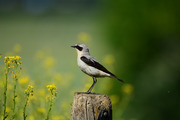

## 1 INTRODUCTION

Habitat quality typically varies both in space and time, and such habitat heterogeneity is expected to have profound impacts on populations' dynamics, persistence and evolution (Liu et al., 2011; Pulliam & Danielson, 1991; Ronce & Kirkpatrick, 2001). This is because habitat quality affects various demographic traits as well as individual movements (Acker et al., 2017; Arlt & Pärt, 2007; Chamberlain et al., 2009; Franklin et al., 2000; Gyllenberg et al., 2016; Low et al., 2010; Rebolo‐Ifrán et al., 2015), potentially causing populations to display a pattern of source–sink dynamics (Pulliam, 1988). Here, source habitats produce a net surplus of individuals (i.e. they have a per capita contribution to the metapopulation > 1; Heinrichs et al., 2016; Runge et al., 2006) and will be the main contributors for self‐sustaining populations. Sinks, by contrast, produce a deficit (i.e. contribution < 1) that can be partly compensated for by immigration (Hixon et al., 2002; Pulliam & Danielson, 1991), and may provide a buffer/reservoir of individuals under temporally variable environmental conditions (Holt, 1997; Howe et al., 1991). Assessing the source–sink status and dynamics of populations is therefore of major importance for understanding population dynamics and for informing decisions concerning the management of natural populations and species (e.g. Crowder et al., 2000; Gaona et al., 1998; Naranjo & Bodmer, 2007; Nielsen et al., 2006).

Despite a multitude of studies over the past 25 years, in‐depth assessments of the source or sink status of habitats for populations have proven extremely difficult (reviewed in Furrer & Pasinelli, 2016). First, habitat quality likely varies in time (Johnson, 2004; Loreau et al., 2013), resulting in temporal variation of source–sink dynamics (Furrer & Pasinelli, 2016; Loreau et al., 2013) and hence difficulties in assessing the net contribution of different habitats and the conditions under which they may shift from source to sink. Second, study areas are often limited in space and rarely cover the full distribution of the study population. Thus, in most cases, it is highly challenging to distinguish mortality from permanent emigration and obtain accurate estimates of surpluses or deficits of individuals produced per habitat or population (Runge et al., 2006). This is true at the scale of the habitat‐specific subpopulations (exchanges between sources and sinks within the population) and also at larger spatial scales (movements between populations). To date, few studies have accounted for permanent emigration when estimating the source or sink status of single study populations (but see Fay et al., 2019; Weegman et al., 2016). Moreover, few studies have considered movements between habitat types or subpopulations in order to better estimate the demographic contribution of local habitats to the overall studied population (but see Paquet et al., 2019; Pasinelli et al., 2011; Seward et al., 2019) and none of these studies accounted for permanent emigration from the study area (Furrer & Pasinelli, 2016). Ignoring permanent emigration out of the study population results in an underestimation of survival in open populations and therefore an overestimation of the presence of sinks (Pasinelli et al., 2011). Furthermore, habitat characteristics likely influence both survival and movement decisions or distances (Acker et al., 2017; Arlt & Pärt, 2008; Low et al., 2010). Thus, local habitat effects need to be accounted for when determining both survival and movement distances. A failure to do so will produce biased estimates of habitat differences in per capita contribution to the population, and hence incorrect assessments of habitat‐specific source–sink dynamics.

One potential solution to these issues is to use integrated population model (IPM; Plard et al., 2019; Zipkin & Saunders, 2018) as they allow simultaneous estimation of all demographic rates necessary to estimate temporal variation in habitat‐specific contributions (Paquet et al., 2019). Spatially explicit IPMs can further take spatial variation in demographic rates into account (Chandler & Clark, 2014; Chandler et al., 2018). By incorporating spatial capture–recapture models that model movement distances from data collected at the local scale of the study area (Schaub & Royle, 2014), such models allow adjustment of the apparent survival estimate for permanent emigration events outside of the study area resulting from these local movements. Thus, such an approach has been recommended to reduce biases in the study of source–sink dynamics but has yet to be applied in this context. To assess the habitat‐dependent source–sink dynamics of a population, we combined a multi‐event IPM (Pradel, 2005; based on previous work in Paquet et al., 2019) with spatial capture–recapture models (Schaub & Royle, 2014). Apparent survival estimates of offspring and adults in two contrasting habitats were adjusted by accounting for juvenile and adult movements. This allowed us to examine the impact of the adjusted survival rates on our inference regarding the source–sink dynamics of a population.

We modelled source–sink dynamics by using 24 years of movement and habitat‐specific demographic data of a farmland passerine, the northern wheatear *Oenanthe oenanthe*. In this population, habitats are distinct due to agricultural land use falling into two broad categories defined by their vegetation structure: ‘Short’ habitats with permanently short or sparse ground vegetation during the breeding season and ‘Tall’ habitats with ground vegetation growing tall during the spring (Pärt, 2001). The habitats occur in a mosaic mix at the landscape scale with Short patches intermingled by Tall ones (Appendix Figure A3 1). Both reproductive success (Pärt, 2001; Tye, 1992) and apparent survival (Arlt et al., 2008; Low et al., 2010) are generally lower in Tall habitats and such habitats act as apparent sinks, whereas Short habitat status is less clear and likely varies over time (Arlt et al., 2008; Paquet et al., 2019). The assessment of source–sink dynamics in this population is particularly likely to be elucidated by the use of a spatial IPM for two main reasons. First, the population is open to emigration, and therefore, permanent movements from inside to outside the population occur which, if ignored, would result in an underestimation of first‐year and breeder survival rates. Second, movement decisions and distances likely vary with habitat type (Arlt & Pärt, 2008; Paquet et al., 2019), potentially biasing habitat‐specific differences in survival. In our previous studies, we have not fully evaluated the source–sink structure of our population as habitat‐specific movement distances were not considered (Paquet et al., 2019). Thus, using adjusted survival estimates together with habitat‐specific reproductive data should allow for more accurate assessments of how the two habitats (and therefore their source or sink status) contribute to the total population and how their contribution varies with time.

To quantify the importance of considering habitat‐specific movement distances when assessing source–sink dynamics, we compare estimates from our spatial IPMs with estimates obtained from IPMs that do not account for movement distances. Additionally, as females and males typically differ in their movements and in how their demographic rates vary with habitat, we modelled both sexes to assess whether habitat‐specific demography differed between the sexes.

## 2 MATERIALS AND METHODS

### 2.1 Study species and site

The northern wheatear is a small insectivorous passerine that breeds in Europe, Asia and North America and overwinters in sub‐Saharan Africa. The study area (60 km^2^, Appendix Figure A3 1) is located southeast of Uppsala in southern central Sweden (59°5 N, 17°5 E). Ground vegetation height is an important indicator of habitat quality with short field layers being associated with higher prey availability (Tye, 1992), lower nest predation risk (Pärt, 2001; Schneider et al., 2012) and higher apparent breeder survival (Low et al., 2010) than sites with tall field layers. As a result, Short sites appear to disproportionally contribute to the growth rate of the population compared to Tall sites (Paquet et al., 2019). About 230 territory sites have been occupied by wheatears at least once in the study area since 1993, and 100–180 pairs breed in the area every year. For each territory site, we record habitat type (Short or Tall), the presence of breeding individuals and, when ringed, their identity. Each site is visited several times during the breeding season (from about mid‐April, when the first Wheatears arrive and establish territories and well before egg laying), while the central part of the study area (~40 km^2^, 179 territory sites, 70–90 pairs per year) is more regularly monitored every 3–5 days (for details, see Arlt et al., 2008; Low et al., 2010). Locations of territory sites were very stable across years and treated identically when territories overlapped by more than two‐thirds (Arlt & Pärt, 2007). We use the *x* and *y* coordinates of the centre of each of those locations (territory sites) as the spatial reference of the sightings (expressed in km).

### 2.2 Demographic data

We used data collected from 1993 to 2016 on population counts, breeding success (sites where successful reproduction was observed in a given year, that is, successful breeding with at least one fledged young, including re‐nesting attempts, vs. failed), number of fledglings and resightings of individuals as breeders, marked as nestlings or breeders in previous years. We refer to successful sites rather than successful nests because when a first breeding attempt fails, breeders may re‐nest at the same territory site. In such case, the first nest is not successful but the second nest may be and if so, the breeding site was considered successful. To estimate habitat‐specific parameters, we used slightly different data subsets. Count data (number of breeding territories occupied) came from a subsample of sites in the central 40‐km^2^ area for which occupancy and field layer height were known every year (*N* = 124 territory sites). Resighting data came from individually colour‐ringed juveniles that fledged and adults that bred anywhere in the total 60‐km^2^ area at sites for which we had information on field layer height at their site of first capture (*N* = 6,278 fledglings, 883 female breeders and 791 male breeders). Data on breeding success and number of fledglings came from the total area from sites for which we had information on field layer height (*N* = 76–168 sites per year with known breeding success and *N* = 31–100 successful sites per year with known number of fledglings). More details on data collection and selection criteria can be found in Paquet et al. (2019).

### 2.3 Integrated population model

We combined the population data on the number of breeding territories with the three different types of demographic data (breeding success, number of fledglings and mark–resightings, including *x* and *y* location data) into an IPM (Besbeas et al., 2002; Schaub & Abadi, 2011) to jointly estimate demographic parameters and derived quantities in order to assess the source–sink status of the habitats. We start by describing the overall population model resulting from our IPM, and then go into the details of how the parameters of the model are informed by the different datasets.

Our model describes a population of breeders in two different habitat types: Short or Tall sites. A deterministic simplification of the model can be described as

$${\left[\begin{array}{c} {\text{NB}}_{\text{Short}}\\ {\text{NB}}_{\text{Tall}} \end{array}\right]}_{t+1}\;=\;A_{t}\;\times \;{\left[\begin{array}{c} \begin{array}{c} {\text{NB}}_{\text{Short}}\\ {\text{NB}}_{\text{Tall}} \end{array} \end{array}\right]}_{t}\;+\;{\left[\begin{array}{c} \begin{array}{c} {\text{Im}}_{\text{Short}}\\ {\text{Im}}_{\text{Tall}} \end{array} \end{array}\right]}_{t+1}$$

where NB_Short_ and NB_Tall_ are the number of breeders on Short and Tall sites and Im_Short_ and Im_Tall_ are the number of net immigrants on Short and Tall sites. Net immigrants are the residual number of individuals that are not predicted by the estimated local vital rates and population structure (Altwegg et al., 2014; Paquet et al., 2019). The matrix $A_{t}$ is the following 2 × 2 Leslie matrix:

$$\left[\begin{array}{cc} b_{S,t}f_{S,t}\varphi_{fl,S,t}\left(1\;‐\;\psi_{fl,S}\right)\;+\;\varphi_{br,S,t}\left(1\;‐\;\psi_{br,S}\right) & b_{T,t}f_{T,t}\varphi_{fl,T,t}\psi_{fl,T}\;+\;\varphi_{br,T,t}\psi_{br,T}\\ b_{S,t}f_{S,t}\varphi_{fl,S,t}\psi_{fl,S}\;+\;\varphi_{br,S,t}\psi_{br,S} & b_{T,t}f_{T,t}\varphi_{fl,T,t}\left(1\;‐\;\psi_{fl,T}\right)\;+\;\varphi_{br,T,t}\left(1\;‐\;\psi_{br,T}\right) \end{array}\right]$$

where *b_S_*_,_ *_t_* and *b_T_*_,_ *_t_* are the yearly breeding success on Short and Tall sites, *f_S_*_,_ *_t_* and *f_T_*_,_ *_t_* are the yearly number of fledglings of each sex (assuming even sex ratio) given success of breeding on Short and Tall habitats, respectively, *φ_fl_*_,_ *_S_*_,_ *_t_* and *φ_fl_*_,_ *_T_*_,_ *_t_* are the yearly survival rate of fledglings born in Short and Tall sites and $\psi_{fl,S}$ and $\psi_{fl,T}$ are their respective probability to breed in the habitat that they were not born in (i.e. $\psi_{fl,S}$ is the probability of yearling breeders born in Short habitats to breed in Tall habitats and vice versa), $\varphi_{br,S,t}$ and $\varphi_{br,T,t}$ are the yearly survival rate of breeders in Short and Tall habitats and $\psi_{br,S}$ and $\psi_{br,T}$ are their respective probability to breed in the other habitat than their previous breeding habitat.

This model only considers one adult age class (cf. Paquet et al., 2019 who allowed parameters to differ between first‐year and older breeders). We made this assumption because our previous model revealed no clear difference in age structure between the two habitats and because the spatial component adds complexity to our model. Furthermore, actuarial senescence is unlikely to affect the source–sink dynamics in our analyses as ≤5% of all breeders are older than 4 years (unpubl. data), the age when actuarial senescence becomes evident for species with a similar life spans (see Nussey et al., 2013). Ignoring age differences halves the number of parameters to estimate while increasing sample size (as age cannot always be assessed, particularly so for females).

#### 2.3.1 Breeding success and number of fledglings

We modelled reproductive output as a two‐step process with the first step being breeding success or failure, and the second step being the number of fledglings produced from successful sites.

The number of successful sites (defined as sites where successful reproduction was observed) counted each year for a given habitat **h** (Short or Tall) was assumed to follow a binomial distribution with *B_h_*_,_ *_t_* ~ Bin(*b_h_*_,_ *_t_*, *R_h_*_,_ *_t_*) with *R_h_*_,_ *_t_* the number of monitored sites in habitat **h** for which the breeding success was known and *b_h_*_,_ *_t_* is the estimated breeding success in habitat **h** this year.

The total number of fledglings from successful sites each year and in each habitat, *F_ht_*, must, by definition, be at least as large as the number of successful sites that they stem from, *S_ht_* (note that *S_ht_* is not identical to *B_h_*_,_ *_t_* since we only included sites from which the number of fledglings was known in this part of the analysis). In order to use a Poisson distribution, which has support for any number ≥0, for the number of fledglings, we first subtracted the number of successful sites. In other words, we modelled *F_h_*_,_ *_t_* − *S_h_*_,_ *_t_*, which is a number ≥0, using a Poisson distribution:

$$F_{h,t}\;‐\;S_{h,t}\;∼\;\text{Poisson}\left(2f_{h,t}S_{h,t}\;‐\;S_{h,t}\right)$$

where 2*f_h_*_,_ *_t_* is the expected number of fledglings per site in habitat **h** and year **t** (two times the number of fledglings of the modelled sex assuming an even sex ratio). *S_h_*_,_ *_t_* was subtracted in the mean of the Poisson distribution to compensate for subtracting it from the total number of fledglings. Parametrizing the number of fledglings as conditional on successful breeding induced no evidence for lack of fit (Paquet et al., 2019, Appendix 2). We modelled the logit of breeding success and the log of the number of fledglings by successful broods with an intercept and random year variation.

#### 2.3.2 Survival

We modelled annual capture–mark–resighting data and location data using a spatial multi‐event model. The spatial model takes habitat‐specific permanent emigration due to inter‐annual local movements (natal and breeding dispersal) into account when estimating habitat‐specific survival.

The state process describes survival and transition probabilities from the two habitat types. Transitions of each individual *i* from state *z_i_*_,_ *_t_* to state *z_i_*_,_ *_t_*_+1_ the following year were set to follow a categorical distribution.

$$z_{i,t+1}|z_{i,t}\;∼\;\text{Categorical}(\Omega_{z_{i},t,}1\ldots 3,i,t)$$

$$\Omega_{z_{i,t},1\ldots 3,i,t}\;=\;\left(\begin{array}{ccc} \varphi_{a_{i,t},S,t}(1\;‐\;\psi_{a_{i,t},S}) & \varphi_{a_{i,t},S,t}\psi_{a_{i,t},S} & 1\;‐\;\varphi_{a_{i,t},S,t}\\ \varphi_{a_{i,t},T,t}\psi_{a_{i,t},T} & \varphi_{a_{i,t},T,t}(1\;‐\;\psi_{a_{i,t},T}) & 1\;‐\;\varphi_{a_{i,t},T,t}\\ 0 & 0 & 1 \end{array}\right)$$

where state 1 is alive in Short habitat, state 2 is alive in Tall habitat and state 3 is dead, the subscript **a_i,t_** for survival and transition probabilities corresponds to the age of the individual *i* at time *t* (fledgling or breeder) and $\Omega_{z_{i,t},1\ldots 3,i,t}$ is the probability matrix for each transition.

The movement process defines the *x* and *y* locations of an individual *i* in year *t *+ 1,

$$G_{i,t+1}\;=\;\left(\begin{array}{c} Gx_{i,t+1}\\ Gy_{i,t+1} \end{array}\right)$$

as a random walk following a student T distribution for *x* and *y* axis, described by three parameters.

$$G_{i,t+1}\;∼\;T(G_{i,t},\sigma_{Gi,t},5)$$

We chose to use a T distribution as it has been shown to best fit data in previous studies of similar study systems (Reidy et al., 2018; Schaub & Royle, 2014) and seemed compatible with observed dispersal movements within the study area (Appendix Figure A3 1–3). The mean parameter $G_{i,t}$ is the location vector of the individual *i* in year *t*. The variance parameter $\sigma_{Gi,t}$ describes the movement variance and this parameter was allowed to vary with habitat types and age as distances to first breeding sites were expected on average longer (natal dispersal) than distances between consecutive breeding sites (breeding dispersal). This variance parameter was set to be identical in the *x* and *y* directions. The third parameter (i.e. degree of freedom) allows for long‐tailed movement distances (Reidy et al., 2018; Schaub & Royle, 2014) and was set to be constant with a value of 5 across habitats and ages for convergence/identifiability reasons. In addition, because breeders tend to stay at the same breeding site more than expected according to a T distribution, we also modelled site fidelity as a Bernoulli process where the probability to not move at all ***Pstay_h_*** depends on habitat type **h**, according to:

$$stay_{i,t}∼\text{Bern}(Pstay_{h})$$

$$\sigma_{Gi,t}\;=\;\left\{\begin{array}{cc} 0 & ,stay_{i,t}\;=\;1\\ \sigma {\text{move}}_{a,h} & ,stay_{i,t}\;=\;0 \end{array}\right\}$$

*Pstay*_,_ *_h_* was set to zero for fledglings. Therefore, both site fidelity of breeders *Pstay*_,_ *_h_* and movement variances of breeders that moved $\sigma {\mathrm{move}}_{br,h}$ and fledglings $\sigma {\mathrm{move}}_{fl,h}$ were allowed to vary with habitat type. The reason for this was that, after breeding, breeders prospect more often close to their breeding sites when breeding in Short sites (Arlt & Pärt, 2008) and, as a result, are also more likely to breed close to their breeding sites the following year when breeding in Short than in Tall sites. As juveniles often move together with their parents after fledging, their movement distances (natal dispersal) may also be affected by habitat type. Although our study site is a mosaic of Short and Tall habitats, we therefore allowed the movement distances of juveniles and breeders to vary with habitat. All movement parameters were set to be constant across years. We modelled the logit of survival rates with an intercept and random year variation while transition probabilities were set to be constants through time for computational reasons.

The observation process links the three true states **z** *_i_*_,_ *_t_*_+1_ (alive breeding in Short sites, alive breeding in Tall sites and dead) and the four observed states **y** *_i_*_,_ *_t_*_+1_ (i.e. events: seen in Short sites, seen in Tall sites, seen but habitat type undetermined and not seen) via a categorical distribution and the transition matrix $\theta_{z_{i,t+1},1\ldots 4,i,t}$ accounting for imperfect detection and undetermined states. The state was considered as undetermined in rare cases when field notes on habitat type were missing or when an individual attempted to breed more than once (successfully or not) in the two types of habitats.

$$y_{i,t+1}|z_{i,t+1}\;∼\;\text{Categorical}(\theta_{z_{i,t+1}},1\ldots 4,i,t)$$

$$\theta_{z_{i,t+1},1\ldots 4,i,t}\;=\;\left(\begin{array}{cccc} p_{i,t}c_{i,t} & 0 & p_{i,t}\left(1\;‐\;c_{i,t}\right) & 1\;‐\;p_{i,t}\\ 0 & p_{i,t}c_{i,t} & p_{i,t}\left(1\;‐\;c_{i,t}\right) & 1\;‐\;p_{i,t}\\ 0 & 0 & 0 & 1 \end{array}\right)$$

Detection probability, ***p_i_*_,_*_t_***, and the probability that the breeding habitat of an observed individual is known, ***c_i_*_,_*_t_***, were set to be constant across years for each sex but were allowed to vary between the central 40‐km^2^ area and the peripheral area (i.e. in the 60‐km^2^ study area but outside the central area where monitoring effort is less intense). Detection probability outside of the 60‐km^2^ study area was set to zero (see Appendices 1 and 4). To demonstrate the importance of considering movement distances using the spatially explicit location data for the estimates of survival, contributions and net immigration (see below), we also ran IPMs that did not account for movement distances (i.e. not using the spatially explicit location data). Consequently, detection probability and state certainty did not vary in space in these models.

#### 2.3.3 Count data

Population counts in Short and Tall sites were modelled using state space models with the above‐mentioned population models describing the underlying population process. To account for demographic stochasticity, we modelled the numbers of locally produced breeding birds using Poisson and Binomial distributions (see Appendices 1 and 4). We accounted for observation errors by linking counts of breeders in Short and Tall habitats to the underlying state process using Poisson distributions.

$${\left[\begin{array}{c} C_{\text{Short}}\\ C_{\text{Tall}} \end{array}\right]}_{t}\;∼\;\text{Pois}{\left[\begin{array}{c} {\text{NB}}_{\text{Short}}\\ {\text{NB}}_{\text{Tall}} \end{array}\right]}_{t}$$

### 2.4 Derived and additional parameters

#### 2.4.1 Contribution

To estimate how much each habitat type added to the total population, we calculated habitat‐specific per capita contribution (C sensu Runge et al., 2006) as:

C*_h_*_,_ *_t_* = *b_h_*_,_ *_t_* × *f_h_*_,_ *_t_* × $\varphi_{fl,h,t}$ + $\varphi_{br,h,t}$, that is, it is the expected number of first‐year recruits from a breeding individual in habitat h plus the survival rate of breeders in that habitat. This represents the expected number of individuals stemming from a breeder (including itself) in habitat *h* in year *t* that are still alive in the total population in year *t *+ 1. Habitat *h* is then defined as a source in year *t* if C*_h_*_,_ *_t_* > 1, and as a sink if C*_h_*_,_ *_t_* < 1. With this definition, the source–sink status of a habitat does not depend on immigration and accounts for emigration since survival of emigrating individuals is considered part of the contribution. A habitat does not have to produce emigrants to be a source and does not have to have immigrants to be a sink (see Runge et al., 2006 for more details).

#### 2.4.2 Net immigration

Our IPM allows estimation of the additional parameters (sensu Riecke et al., 2019) Im_Short_, Im_Tall_. These are estimated independently for each year. One interpretation of them is that they represent, as the name suggests, the net numbers of immigrants in Short and Tall habitats (see also Fay et al., 2019 for a similar formulation). This may be seen by rewriting the population model above as (again omitting demographic stochasticity for clarity):

$${\left[\begin{array}{c} \begin{array}{c} {\text{Im}}_{\text{Short}}\\ {\text{Im}}_{\text{Tall}} \end{array} \end{array}\right]}_{t+1}\;=\;{\left[\begin{array}{c} {\text{NB}}_{\text{Short}}\\ {\text{NB}}_{\text{Tall}} \end{array}\right]}_{t+1}\;‐\;A_{t}\;\times \;{\left[\begin{array}{c} \begin{array}{c} {\text{NB}}_{\text{Short}}\\ {\text{NB}}_{\text{Tall}} \end{array} \end{array}\right]}_{t}$$

Thus, it is the difference between the number of breeders in a given habitat inside the local population (estimated from count data) and the projected total number of breeders in this habitat in the total population originating from the local population in the previous year. A positive net immigration, therefore, indicates that there are more breeders in the habitat in the local population in year *t *+ 1 than the local population in year *t* would be expected to produce. A negative net immigration suggests that the local population in year *t* should give rise to more breeders in the habitat than what was observed locally in year *t *+ 1, suggesting a net outflow of breeders (Appendix Table A1 for an illustration). Presented differently, it corresponds to the difference between the expected number of immigrants (from outside the local population) towards a given habitat and the expected number of emigrants towards this habitat (from within the local population in either habitat).

Since net immigration is an additional parameter (Riecke et al., 2019), it is not directly informed by data. An alternative interpretation of them is therefore that they are residual parameters and may also reflect errors in the model assumptions for any of the component datasets (Riecke et al., 2019; Schaub & Fletcher, 2015, see also Paquet et al., 2019 for discussion regarding the current dataset). For this reason, we are cautious with drawing strong biological conclusions from the ‘net immigration’ parameters.

#### 2.4.3 Emigration

The spatial capture–resighting model allows computing derived estimates of emigration rates by considering movements originating inside the study area but ending up outside it. We provide estimates of such sex‐, age‐ and habitat‐specific emigration rates in Appendix 5.

### 2.5 Model fitting

We constructed Bayesian IPMs inspired by examples in Schaub and Royle (2014) and Paquet et al. (2019) using jags, version 4.2.0 (Plummer, 2015) run using the rjags package (Plummer, 2013) in Program R, version 3.3.2 (R Core Team, 2016). We estimated parameters using vague priors (see Appendix 4 for priors and initial values). Posterior samples from three Markov Chain Monte Carlo (MCMC) chains were based on 30,000 iterations after an adaptation period of 29,000, a burn‐in of 1,000 and thinning interval of 30 (see Appendix 4). We assessed model convergence both visually and by using the ‘R hat’ Gelman–Rubin statistic (Gelman & Rubin, 1992). Although mixing of some of the underlying movement parameters was low (see Appendix 3 for more details and discussion), there were no apparent issues for the demographic parameters of interest. We performed posterior predictive checks for population count, the number of successful sites, the number of fledglings among successful sites and the multi‐event CMR observation and state models, and obtained satisfactory model performance (see Appendix 2). In the result table, we present the means (and 95% CIs) from the posterior distributions of interest and (by convention) we interpret differences between estimates as statistically ‘clear’ when 95% CI did not overlap (Dushoff et al., 2019). We computed *p*(difference > 0) by calculating the difference between posterior samples (for each iteration and each chain) and computing the proportion of positive differences. When comparing estimates from the female and the male models, or models with and without movements, we compared posterior samples of the same chain and iteration number (but they did not represent the *same* chain and iteration as these models were run separately).

## 3 RESULTS

### 3.1 Habitat‐specific demographic estimates

**Figure 1:**
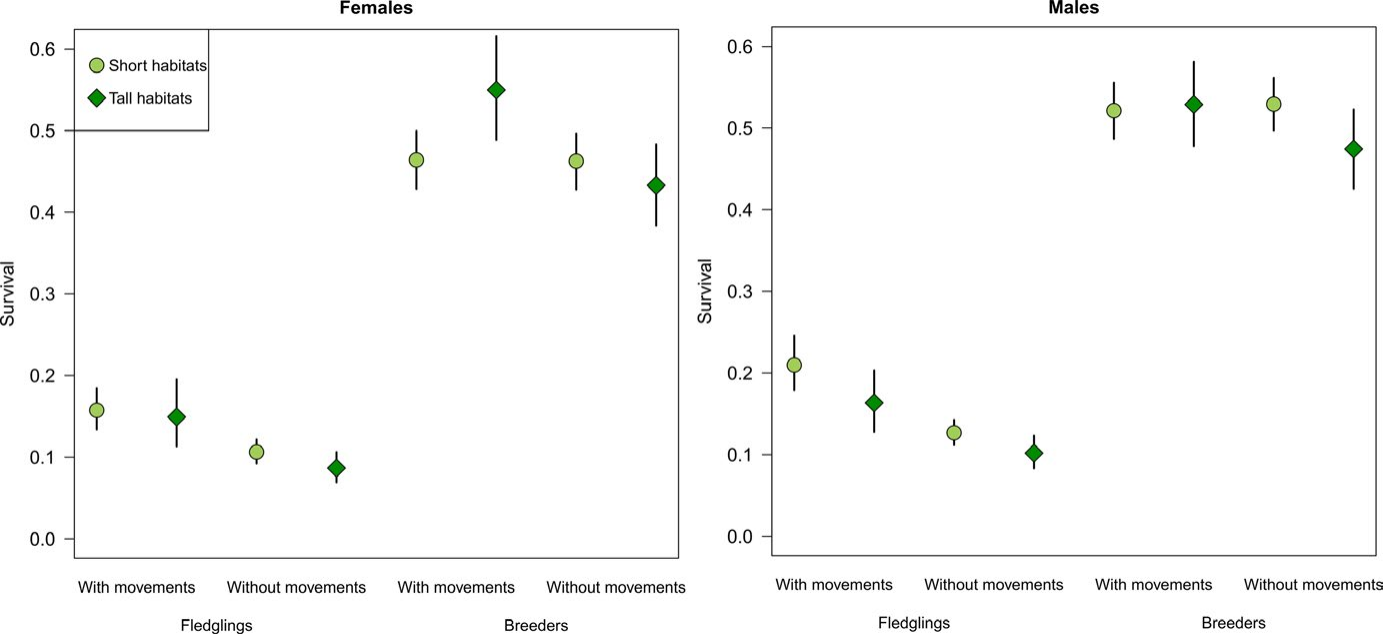
Habitat‐specific estimates (means and 95% credible intervals) of first‐year and breeding survival rates for females (left panel) and males (right panel) with and without accounting for emigration as estimated from observed local movements, that is, from spatially explicit versus non‐explicit integrated population models

Our spatial IPM estimated survival as similar for female fledglings born in Short and Tall habitats while male fledglings from Short habitats had higher mean survival than those from Tall habitats (*p*(difference > 0) = 0.96; Table 1; Figure 1). In short habitats, estimated survival tended to be higher for male fledglings than for female fledglings (*p*(difference > 0) = 0.99; Table 1, Figure 1). Overall, estimated survival of fledglings was clearly higher in spatial than in non‐spatial IPMs (Figure 1). For breeders, females breeding in Tall habitats tended to have higher survival rates than females in Short habitats (*p*(difference > 0) = 0.98), but this trend was not found for males (Table 1; Figure 1). Contrary to the results for fledglings, estimated breeding survival was not clearly different between spatial and non‐spatial IPMs, except for females breeding in Tall habitats whose survival was clearly higher when using the spatial IPM (*p*(difference > 0) = 0.996; Figure 1). Both breeding success and number of fledglings from successful broods were higher in Short than in Tall habitats (Table 1).

**Table 1:** Estimated arithmetic means of demographic parameters for females (**F**) and males (**M**) with associated 95% Bayesian credible intervals (BCI) from the spatially explicit integrated population model (IPM). **N**: the number of breeding pairs in each habitat type, **b**: breeding success, **f**: number of fledglings at successful sites, **φ**: breeder (*br*) and first‐year (*fl*) survival probabilities, Ω: net immigration rates (the number of net immigrants to the focal habitat divided by the total number of breeders in this habitat the same year), **Short** and **Tall**: sites with Short or Tall ground vegetation. All parameters were estimated from the spatially explicit IPMs, that is, accounting for emigration as estimated from observed local movements. Temporal variances are given in Appendix Table A2

Parameter	Mean (95% BCI)
***N*_Short_**	47.59 (44.92, 50.42)
***N*_Tall_**	27.72 (25.75, 29.83)
***b*_Short_**	0.79 (0.77, 0.81)
***b*_Tall_**	0.65 (0.62, 0.68)
***f*_Short_**	2.83 (2.76, 2.91)
***f*_Tall_**	2.45 (2.35, 2.55)
**φ*_fl_*_Short F_**	0.16 (0.13, 0.18)
**φ*_fl_*_Tall F_**	0.15 (0.11, 0.20)
**φ*_fl_*_Short M_**	0.21(0.18, 0.25)
**φ*_fl_*_Tall M_**	0.16 (0.13, 0.20)
**φ*_br_*_Short F_**	0.46 (0.43, 0.50)
**φ*_br_*_Tall F_**	0.55 (0.49, 0.62)
**φ*_br_*_Short M_**	0.52 (0.49, 0.56)
**φ*_br_*_Tall M_**	0.53 (0.48, 0.58)
**Ω_Short F_**	0.13 (0.02, 0.22)
**Ω_Tall F_**	0.12 (−0.04, 0.26)
**Ω_Short M_**	0.05 (−0.06, 0.14)
**Ω_Tall M_**	−0.08 (−0.27, 0.08)

### 3.2 Assessing source–sink status: Habitat‐specific contributions and their temporal variation

**Figure 2:**
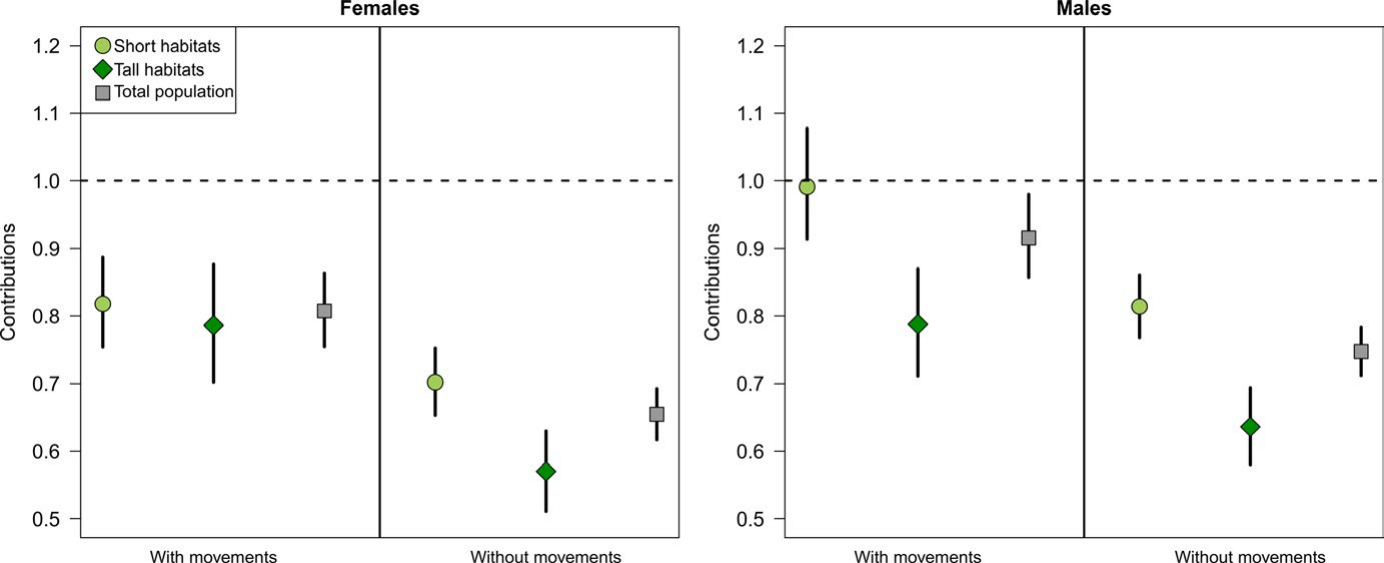
Per capita contribution (means and 95% CIs) of Short sites (light green circles), Tall sites (dark green diamonds) and the overall population (grey squares) with and without accounting for emigration as estimated from local movements. See main text for definitions and details on calculations. Contributions lower than one indicate sink habitats and population while values higher than one indicate source habitats and population

Although contributions were sensibly increased when using spatial IPMs, both Short and Tall habitats still produced a deficit of females and acted as similar sinks (Figure 2).

For males, the spatial IPM revealed that Short habitats may act as a source while Tall habitats remained sinks (Figure 2).

**Figure 3:**
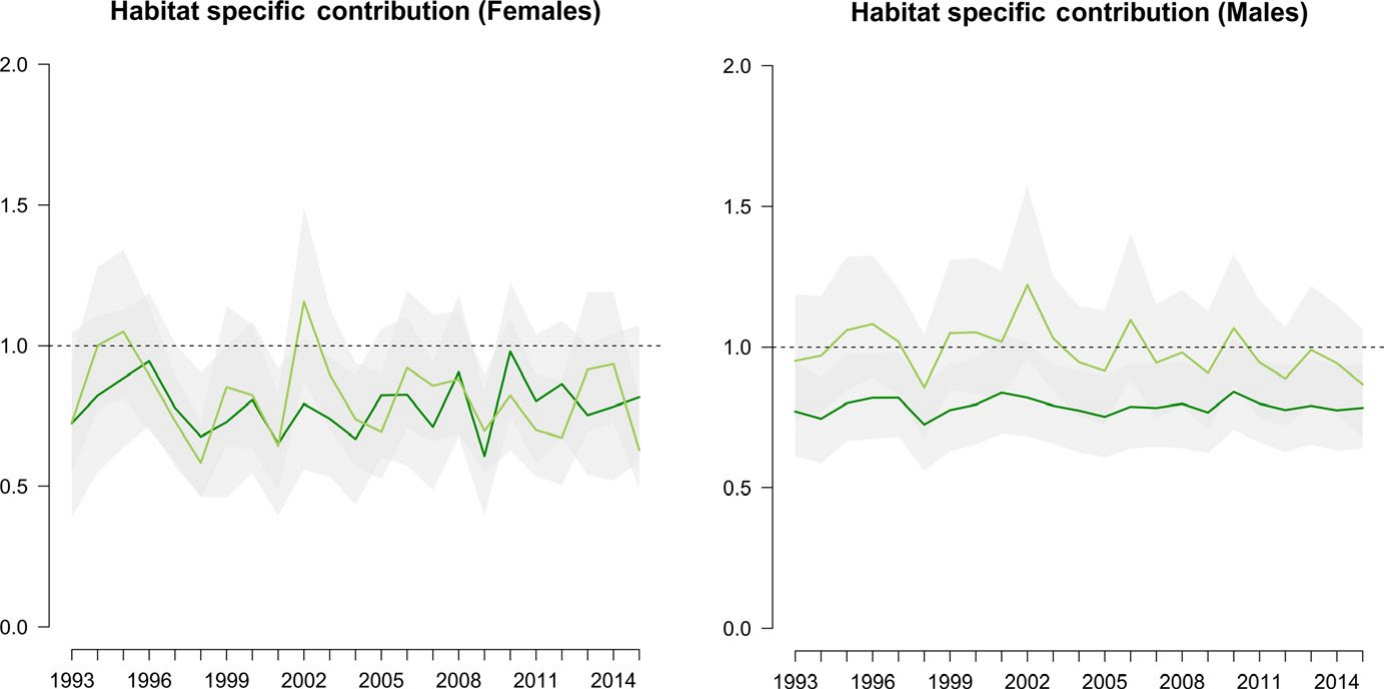
Per capita contribution of Short sites (light green) and Tall sites (dark green) across years (means and 95% CIs) for females (left panel) and males (right panel) after accounting for emigration as estimated from local movements. Contributions lower than one indicate sink habitats while values higher than one indicate source habitats

Over time, no habitat clearly acted as source of females, and both acted as distinct sinks some years (Figure 3). Contribution never distinctly differed between habitats (i.e. 95% CI of the annual differences in contribution always overlapped zero). In contrast, for males, there were clearer differences between the habitats. Short habitats never acted as distinct sinks while Tall habitats did so in nearly all years (Figure 3). Annual contribution was on average higher in Short than in Tall sites (Figure 3), and showed distinct differences in 6 of 23 years.

### 3.3 Net immigration rates and emigration rates

**Figure 4:**
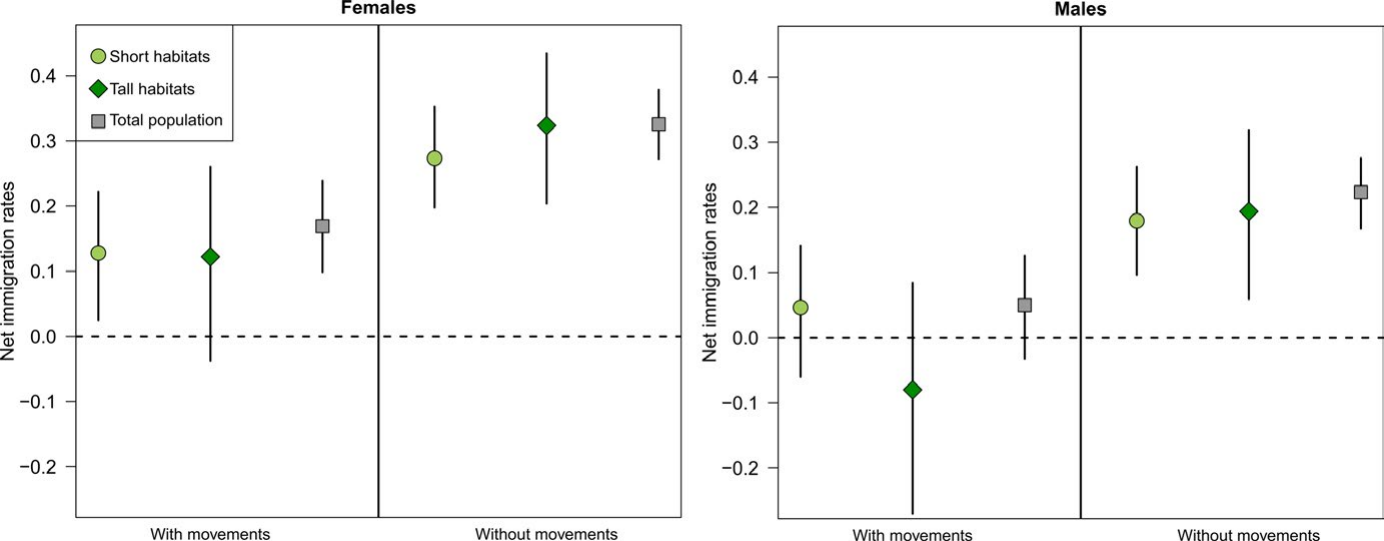
Net immigrations rates (means and 95% CIs) of Short sites (light green circles), Tall sites (dark green diamonds) and the overall population (grey squares) calculated as the number of net immigrants to the focal habitat divided by the total number of breeders in this habitat the same year, with and without accounting for emigration as estimated from local movements

For females, net immigration was estimated to be positive and similar in both habitats (Figure 4). For males, credible intervals for net immigration overlapped zero (Figure 4). Estimated emigration rates largely mirrored the differences in survival rates between the spatial and non‐spatial IPMs discussed above. Specifically, estimated emigration rates of breeders were low in Short habitats, but substantially larger than zero in Tall habitats, at least for females, and emigration rates of fledglings were generally higher than for breeders (Appendix Figure A5 1–4).

## 4 DISCUSSION

Assessing the source–sink status and dynamics of habitats and populations is of prime importance for our understanding of the dynamics and ecology of populations (Liu et al., 2011). In their review, Furrer and Pasinelli (2016) recommended that source–sink assessments should be based on (a) all demographic parameters including emigration/immigration, (b) better estimates of survival by using spatially explicit models and (c) an assessment of the temporal dynamics of source and sink habitats when possible. Here, we built a spatial IPM to fulfil all these recommendations. Moreover, because males and females often differ in demographic rates and movement distances, we added sex‐specific estimates to assess whether habitat‐specific demography differed between the sexes. Our results demonstrate that taking into account local movements and sex differences can sensibly affect source–sink assessments of habitats and populations, and estimated habitat differences in per capita contribution to the population. And while we did not find evidence for temporal changes in whether a habitat acted as source or sink, the relative difference in contribution between habitats varied over time.

When using a spatial IPM in which local movement distances were accounted for, Short (higher quality) habitats were suggested to be self‐sustainable for males, whereas the same was not evident for female wheatears in our study population. When not accounting for movement distances, no habitat for either sex acted as a source. Clearly, ignoring movements out of the study area will lead to underestimating survival rates and therefore also overestimate the presence of sinks (Newby et al., 2013), which are, perhaps consequently, more often reported than sources in previous studies (Furrer & Pasinelli, 2016). We found that including natal, in comparison to breeding, local movements were mostly responsible for the increase in estimated per capita contributions, which is not surprising given that natal dispersal is on average more extensive than breeding dispersal (Greenwood & Harvey, 1982, see Appendix 3 Figure A3 2). As such movements are often covering long distances, using the method of Schaub and Royle may only be possible when study areas are large enough to capture natal dispersal distributions (Schaub & Royle, 2014). Therefore, as study areas are always limited in size, there still exists some uncertainty in the true patterns of natal dispersal (see below). That being said, using information from local movements from large study areas, and most particularly juvenile natal dispersal, will provide higher and potentially more accurate estimates of contribution of habitats and populations.

For female wheatears, we found no clear habitat differences in per capita contribution when habitat‐specific movements were accounted for. This is because female juveniles originating from, and females breeding in, Tall (lower quality) habitats on average moved longer local distances between years than those originating in Short habitats in our study population (see Figures A3 2 and A3 3). If this habitat‐specific difference in local movements in females reflects a corresponding difference at a larger scale (out of the study area), then there is also a difference in the degree of underestimation of local survival for females. In our case, underestimation of local survival would be more pronounced in Tall habitats. Hence, habitat differences in survival (and consequently contribution) can be biased when habitat‐specific movements are ignored. Habitat differences in movements are commonplace in natural populations (Byholm et al., 2003; Kenward et al., 2001). Our results therefore strongly encourage future studies to consider habitat differences in movements (dispersal) when estimating habitat differences in survival.

In our case study, we found that Short habitats may act as sources for males while both Short and Tall habitats were estimated to be sinks for females. That one habitat type may act as sources for one sex but as sinks for the other sex requires some explanation. One possibility is that true survival is indeed higher for males than for female wheatears, hence causing the contribution of the male part of the population breeding in short habitats to reach a source status. In accordance with this hypothesis, adult sex ratio is typically male biased in birds (Donald, 2007) partly because of increased predation of breeding females (Post & Götmark, 2006a, 2006b; Low et al., 2010; for additional reasons for female‐biased mortality, see Donald, 2007). Another hypothesis is that a larger fraction of permanent emigration events that is not captured by the modelled local movements occurs among females. This would lead to a stronger underestimation of true survival for females than for males even after accounting for local movements. In line with this hypothesis, breeding and natal dispersal movements are typically female biased in birds (Greenwood & Harvey, 1982; Végvári et al., 2018). As almost all empirical data come from limited study areas, it remains challenging to know how well distances estimated from movements within study areas reflect movements out of the study areas. Consequently, Schaub and Royle's spatial CMR model has to make assumptions concerning dispersal patterns (e.g. choice of the dispersal distribution, movements independent of location) which, if they are not good approximations of true movements, could result in biased estimates of survival and emigration rates. The fact that we found no tendency for longer natal dispersal of female wheatears within our study area (which is otherwise commonly observed in birds) might therefore reflect effects of a limited study area even though our study area is relatively large. By extension, an absence of a female‐biased movement distances *within* the study area cannot be taken as evidence for an absence of female‐biased longer distance movements *outside* the study area. Clearly, there is a strong need for more studies investigating the relationships between local and longer distance movements in order to make realistic assumptions about movements and elucidate whether the sexes differ in their response to habitat quality.

Results from our spatial IPM have important implications for our understanding of the ecology and demography of our study population. First, it confirms the assumption that Short habitats such as grazed pastures are indeed ‘good’ habitats for wheatears that may act as sources for males, which could not be confirmed when using non‐spatial IPMs (see also Paquet et al., 2019). Second, the spatial IPM suggests that females survive slightly better when breeding on Tall habitats than on Short habitats (as a consequence of their higher estimated emigration rates) while our previous results based on nest predation risk, nest predation and foraging effort when feeding nestlings of females suggest the opposite (Low et al., 2010; Pärt, 2001). Clearly, these contradictory results call for further investigations not only on dispersal (see conclusion) but also on phenotypic indicators of subsequent survival (e.g. body condition, telomere length) and whether similar indicators are related to dispersal propensity. Finally, we found evidence for differences in movement distances according to habitat quality, age (juvenile vs. breeders) and sex. Such differences likely occur in other populations as well, with potentially important consequences for our understanding of their ecology and demography. More generally, we recommend comparing inferences with and without taking into account movement distances (and keeping in mind their respective assumptions) for a better understanding of species ecology and informed conservation strategies.

## 5 CONCLUSIONS

Assessing source–sink status of habitats and populations has been challenging. Here, we show that spatial IPMs, taking into account permanent emigration using information on local movements, can reduce biases in the per capita contribution of different habitats by reducing the underestimation of survival rates and hence the overestimation of the occurrence of sink habitats (Furrer & Pasinelli, 2016). Since movements outside the study area are usually unknown, it is challenging to know to what degree potential larger scale movements processes may be overlooked (Taylor et al., 2015), and hence, how well the modelled local movement distributions reflect true movement distributions including longer distance movements. An open but very central question is therefore to what extent patterns of short‐distance movements within limited study areas can contribute to estimates of longer distance movements and permanent emigration rates. Several alternative methods can help better estimate movements, such as satellite tracking (Powell et al., 2000), genetic tools (Millon et al., 2019; Peery et al., 2008) and citizen science data (Chadoeuf et al., 2018), all coming with strengths and limitations (Chadoeuf et al., 2018; Koenig et al., 1996). Integrated models offer an exciting opportunity to combine all available data on demographic rates and movements, which will refine our knowledge on movement patterns and allow for more detailed studies of potential sex differences, source–sink dynamics and for improving potential conservation interventions to halt population declines of species at risk.

## AUTHORS' CONTRIBUTIONS

M.P., D.A., T.P., J.K., M.L. and P.F. conceived the study; T.P., D.A., J.K. and M.L. contributed to data collection; M.P. analysed the data with help from J.K. M.P. led the writing of the manuscript together with T.P. and D.A. All other authors contributed to the drafts and gave final approval for submission.

## Supporting information

Supplementary Material

## Data Availability

Data available from the Dryad Digital Repository https://doi.org/10.5061/dryad.v9s4mw6t0 (Paquet et al., 2020).
