# Supplementary material for: Why we should care about movements: Using spatially explicit integrated population models to assess habitat source–sink dynamics
Source: J Anim Ecol. 2020 Oct 20;89(12):2922–33. doi: 10.1111/1365-2656.13357 (PMC7756878; doi:10.1111/1365-2656.13357)
Supplement: Supplementary file 1 — Supplementary Material [file JANE-89-2922-s001.docx]

**Appendix section summary:**

**Appendix 1:** Model description

**Appendix 2:** Post predictive checks of the model

**Appendix 3:** Map of the study area and observed and estimated inter-annual distances

**Appendix 4:** JAGS code to fit the spatially explicit integrated population model (including code for post predictive checking).

**Appendix 5:** Estimated emigration rates and R code used to compute them.

**Table A1:** Illustration of the estimation of net immigration

**Table A2:** Estimated coefficients of temporal variance of the demographic parameters

**Table A3:** Estimates of transition probabilities, resighting probabilities and state certainties.

**Appendix 1: Model description**

***LEGENDS:***

***h*** subscript is the habitat type: h1 or h2 for habitat with short field layer height i.e. Short habitat and habitat with tall or growing field layer height i.e. Tall habitat

***a*** subscript is the age class: fledgling (fl) or breeder/adult (br)

**φ*_br_*** survival of breeders (corrected for permanent emigration due to local movements)

**ψ** transition parameter from habitat *h* to the other

**b** breeding success

**fl** number of fledglings of the modelled sex produced per successful breeding attempt

**φ*_fl_*** first-year survival (corrected for permanent emigration due to local movements)

**ψ*_fl_*** first-year transition parameter from habitat *h* to the other

**I** net immigration rate (can be positive or negative)

**P_l_** re-sighting probability of a breeder (vary with the location **l**: central P***c*** vs peripheral P***p*** study area**)**

**C_l_** state certainty. Probability to know the habitat of a re-sighted breeder (vary with the location **l**: central P***c*** vs peripheral P***p*** study area**)**

**POPULATION COUNTS state space model**

**Observation process:**

C_h,t : observed number of sites occupied in habitat h at time t_

C_,h,t_~Poisson(N_h,t_)

**System process:**

**Number of occupied territory sites (breeding pairs):**

N **_h1_**_,t_ = N*_fl moving from_* **_h2_**_,t-1_+ N*_fl from_* **_h1_**_,t-1_– N*_fl moving from_* **_h1_**_,t-1_ + N*_br moving from_* **_h2_**_,t-1_+ N*_br from_* **_h1_**_,t-1_– N*_br moving from_* **_h1_**_,t-1_+ N_imm_ **_h1_**_, t_

N **_h2_**_,t_ = N*_fl moving from_* **_h1_**_,t-1_+ N*_fl from_* **_h2_**_,t-1_– N*_fl moving from_* **_h2_**_,t-1_ + N*_br moving from_* **_h1_**_,t-1_+ N*_br from_* **_h2_**_,t-1_– N*_br moving from_* **_h2_**_,t-1_+ N_imm_ **_h2_**_, t_

**Number of net immigrants (can be positive or negative) towards each habitat h:**

N_imm h,t_= round(I _h,t_ × N _h,t-1_)

**Number of breeders on each habitat h (Short or Tall) that survived *and* changed breeding habitat type from year t-1 to year t:**

N*_br moving from_* _h,t-1_~Binomial(ψ_h_**,** N*_br survived_* _h,t-1_)

**Number of breeders on each habitat that survived from year t-1 to year t:**

N*_br survived_* _h,t-1_~Binomial(φ_h,t-1_**,** N_h,t-1_)

**Number of fledglings on each habitat h (Short or Tall) that survived *and* changed habitat type (from their natal to their first breeding habitat) from year t-1 to year t:**

N*_fl moving from_* _h,t-1_~Binomial(ψ*_fl_*_,h_**,** N*_fl survived_* _h,t-1_)

**Number of fledglings on each habitat that survived from year t-1 to year t:**

N*_fl survived_* _h,t-1_~Binomial(φ*_fl_* _h,t-1_**,** N*_fl_* _h,t-1_)

**Number of fledglings of the modelled sex on each habitat (we assume sex ratio 1-1):**

N*_fl_* _h,t-1_~Binomial(0.5**,** Nmf*_fledged_* _h,t-1_)

**Nmf*_fl_* _h,t_ is the number of fledglings (males plus females) produced on each habitat**

Nmf*_fle_* _h,t-1_- N *_successful_* _,h,t-1_~Poisson(N *_successful_* _h,t-1_ × (2 × fl _h,t-1_-1))

**fl _h,t_** **is the average number of fledglings of each sex produced by a successful site**

**N *_successful_* _h,t_** **is the number of successful sites of each habitat:**

N *_successful_* _h,t-1_~Binomial(b_h,t-1_, N _h,t-1_)

**b_h,t_ is the breeding success probability for each habitat type a given year.**

**N _h,t-1_ is the number of breeders for each habitat type the year t-1**

**CAPTURE RECAPTURE DATA:**

Multistate multi-event CR model

**z***_i,t_*: True state of individual i at time t

$z_{{i,f}_{i}}$ = $y_{{i,f}_{i}}$= fs_i_ (state of individual i at first encounter)

**z**_i,t+1_|**z**_i,t_~Categorical($\Omega_{z_{i,t},1\ldots3,i,t}$)

$$\Omega_{\boldsymbol{z}_{\boldsymbol{i}\boldsymbol{,}\boldsymbol{t}}\boldsymbol{,}\boldsymbol{1}\boldsymbol{\ldots}\boldsymbol{3}\boldsymbol{,}\boldsymbol{i}\boldsymbol{,}\boldsymbol{t}}=\left( \begin{matrix} \varphi_{a_{i,t},\mathbf{h1},t}{(1-\psi}_{a_{i,t},\mathbf{h1}}) & \varphi_{a_{i,t},\mathbf{h1},t}\psi_{a_{i,t},\mathbf{h1}} & 1-\varphi_{a_{i,t},\mathbf{h1},t} \\ \varphi_{a_{i,t},\mathbf{h2},t}\psi_{a_{i,t},\mathbf{h2}} & \varphi_{a_{i,t},\mathbf{h2},t}{(1-\psi}_{a_{i,t},\mathbf{h2}}) & 1-\varphi_{a_{i,t},\mathbf{h2},t} \\ 0 & 0 & 1 \end{matrix} \right)$$

States:

**1**: alive in Short

**2**: alive in Tall

**3**: dead

**y**_i,t_: Observed state (i.e. event) of individual i at time t

**y**_i,t+1_|**z**_i,t+1_~Categorical($\theta_{z_{i,t+1},1\ldots4,i,t}$)

$\theta_{z_{i,t+1},1\ldots4,i,t}$= $\left( \begin{matrix} p_{i,t}c_{i,t} & 0 & p_{i,t}\left( 1-c_{i,t} \right) & 1-p_{i,t} \\ 0 & p_{i,t}c_{i,t} & p_{i,t}\left( 1-c_{i,t} \right) & 1-p_{i,t} \\ 0 & 0 & 0 & 1 \end{matrix} \right)$

**p** is recapture probability of a breeder and **c** is the probability to know the habitat of the breeder i.e. state certainty

Observed states (events):

1: seen in Short

2: seen in Tall

3: seen but habitat unknown

4: not seen

**LOCATION DATA** (and associated recapture probabilities and state certainties)**:**

$G_{i,t}=\left( \begin{matrix} {Gx}_{i,t} \\ {Gy}_{i,t} \end{matrix} \right)$ Location vector of individual i at time t

$G_{i,fi}={G2}_{i,fi}$ Location at first capture

**Gx**_i,t+1_ ~Normal(**G2x**_i,t+1_, 1.10^-5^)

**Gy**_i,t+1_ ~Normal(**G2y**_i,t+1_, 1.10^-5^)

This parametrisation greatly improved the mixing of the chains

**G2x**_i,t+1_ = **G2x**_i,t_ + eta.x_i,t+1_

**G2y**_i,t+1_ = **G2y**_i,t_+ eta.y_i,t+1_

eta.x_i,t+1_~Student(0, sigmax_i,t+1_,5)

eta.y_i,t+1_~Student(0, sigmay_i,t+1_,5)

Inter-annual location distances on the x and y axis (in km):

sigmax_i,t+1_ = stay_i,t_ × 1.10^-5^ + (1- stay_i,t_) × sigma.x_a,h_

sigmay_i,t+1_ = stay_i,t_ × 1.10^-5^ + (1- stay_i,t_) × sigma.y_a,h_

When a breeder was (or was estimated to be) site-faithful (stay_i,t_=1) then its dispersal variance was set to be extremely narrow (1.10^-5^ km=1cm).

stay_i,t_ ~Bernoulli(probstay_a,h_)

probstay_fl,h_ = 0

probstay_br,h_ ~Uniform(0,1)

sigma.x_a,h_~Uniform(0,3.5)

sigma.y_a,h_~Uniform(0,3.5)

Resighting probability and state certainty:

p_i,t_ = r*_centre_* _i,t_ × p*_c_* + (r*_total_* _i,t_ - r*_centre_* _i,t_ ) × p*_p_*

c_i,t_ = r*_centre_* _i,t_ × c*_c_* + (r*_total_* _i,t_ - r*_centre_* _i,t_ ) × c*_p_*

r*_centre_* _i,t_ = 1 if **G_i,t_** $\in$ **A_c_** : central area state space. Zero if outside the central area.

r*_total_* _i,t_ - r*_centre_* _i,t_ = 1 if **G_i,t_** $\in$ **A*_p_*** : peripheral area state space (total area excluding central area). Zero if outside the peripheral area.

p*_c_*~Uniform(0,1)

p*_p_*~Uniform(0,1)

c*_c_*~Uniform(0,1)

c*_p_*~Uniform(0,1)

**BREEDING SUCCESS DATA:**

**B**_h,t_ :The number of counted successful sites

**R**_h,t_ :The number of sites for which the breeding success is known

**B**_h,t_~Binomial(b_h,t_,R_h,t_)

**NUMBER OF FLEDGLINGS DATA:**

**F**_h,t_ :The number of counted fledglings (among successful sites of known number of fledglings)

**S**_h,t_ :The number of sites for which the number of fledglings is known

**F**_h,t_**-S**_h,t_ ~Poisson(**S**_h,t_ **(2fl**_h,t_-1))

**CONSTRAINS AND OTHER PRIORS**

For **fl**_h,t_ (mean number of fledged males/females for each parent’s habitat)

log(**fl**_h,t_)= mu_h_+ε_h,t_

mu_h_~Normal(0, 1e+06)

ε_h,t_~Normal(0,sigma)

sigma~ Student(0,1,1) T(0,) (Half Cauchy prior)

Net immigration rate:

**I_h,t_** ~Uniform(-2,2)

For **φ*_fl_***_,h,t_ **φ**_br,h,t,_ **b**_h,t_

logit(parameter)= mu_h_+ε_h,t_

mu_h_~Normal(0, 1e+06)

ε_h,t_~Normal(0,sigma)

sigma~ Student(0,1,1) T(0,) (Half Cauchy prior)

For*,* **ψ*_fl_*** _h_ **ψ*_br_***_h_*,*

logit(parameter)= mu_h_

mu_h_~Normal(0, 1e+06)

**Initial values are available from the script file in Appendix 4.**

**Appendix 2: Posterior predictive checks of the model**

The overall goodness of fit of our sub models for i) the **number of successful sites**, ii) the **number of fledglings among successful sites and** iii) **the number of occupied sites** were assessed using the χ² discrepancy metric (Gelman, Meng, & Stern, 1996). This metric compares the distance of the observed data (or partly observed, partly simulated for the location data) to the model (discrepancy of data), calculated as:

$$D_{\chi^{2}}^{obs}=\sum_{h=1}^{2} \sum_{t=1}^{24} \frac{\left( y_{h,t}^{obs}-E\left( y_{h,t} | \theta\right) \right)^{2}}{Var\left( y_{h,t} | \theta\right)}$$

with the distance of “perfect” replicated data to the model (discrepancy of replicate data):

$$D_{\chi^{2}}^{rep}=\sum_{h=1}^{2} \sum_{t=1}^{24} \frac{\left( y_{h,t}^{rep}-E\left( y_{h,t} | \theta\right) \right)^{2}}{Var\left( y_{h,t} | \theta\right)}$$

where $y_{h,t}^{obs}$ and $y_{h,t}^{rep}$represent the observed or replicated data for each habitat, age class and year and θ represents the estimated subset of parameters.

Posterior predictive probabilities (reported as P in the figures below) inform on how many times in our posterior samples the distance of the replicated data to the expected mean is greater than the distance of the observed data to the expected mean. A value of 0.5 suggests a good fit while values close to 0 or 1 (classically below 0.05 or above 0.95) suggest a lack of fit. Here we present results from the female model as figures from the male model were nearly identical.

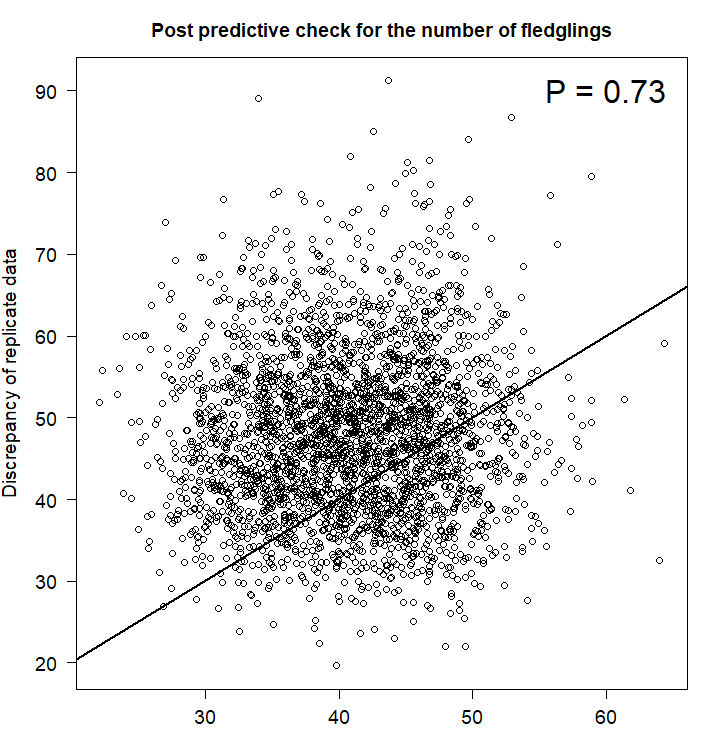

To check the goodness of fit of our **capture recapture models** we assessed the probabilities that the observed events (e.g. four possible events including uncertain state and not re-sighted) matched with events simulated from the categorical likelihoods used to model the observation process given the true states (Acker, 2017; Greenhill, Ward, & Sacks, 2011; Paquet et al., 2019).

In addition, as the latent true states are partly informed by the data on the observed events (e.g. an observed event “captured/re-sighted on short habitat” necessarily leads to the latent state “alive on short habitat”), we also assessed the goodness of fit of the state processes by comparing the partly observed, partly estimated latent state of our model with a latent state 100% simulated from the categorical likelihoods used to model the state process given the model.

These probabilities were only calculated for the states and events following the first year of capture (as states and events at first sighting or before always match) and up to the last year of study.

The proportion of matching events were 0.993 (95% CI 0.992-0.994) for females and 0.992 (0.991-0.994) for males and the proportion of matching states were 0.956 (0.952-0.959) for females and 0.949 (0.944-0.953) for males.

Finally, we performed goodness of fit tests of our CMR model using the package R2Ucare (Gimenez, Lebreton, Choquet, & Pradel, 2018) that suggested no lack of fit.

JAGS code to assess goodness-of-fit is included in the script in **Appendix 4.**

**Appendix 3: Observed and estimated inter-annual location distances**


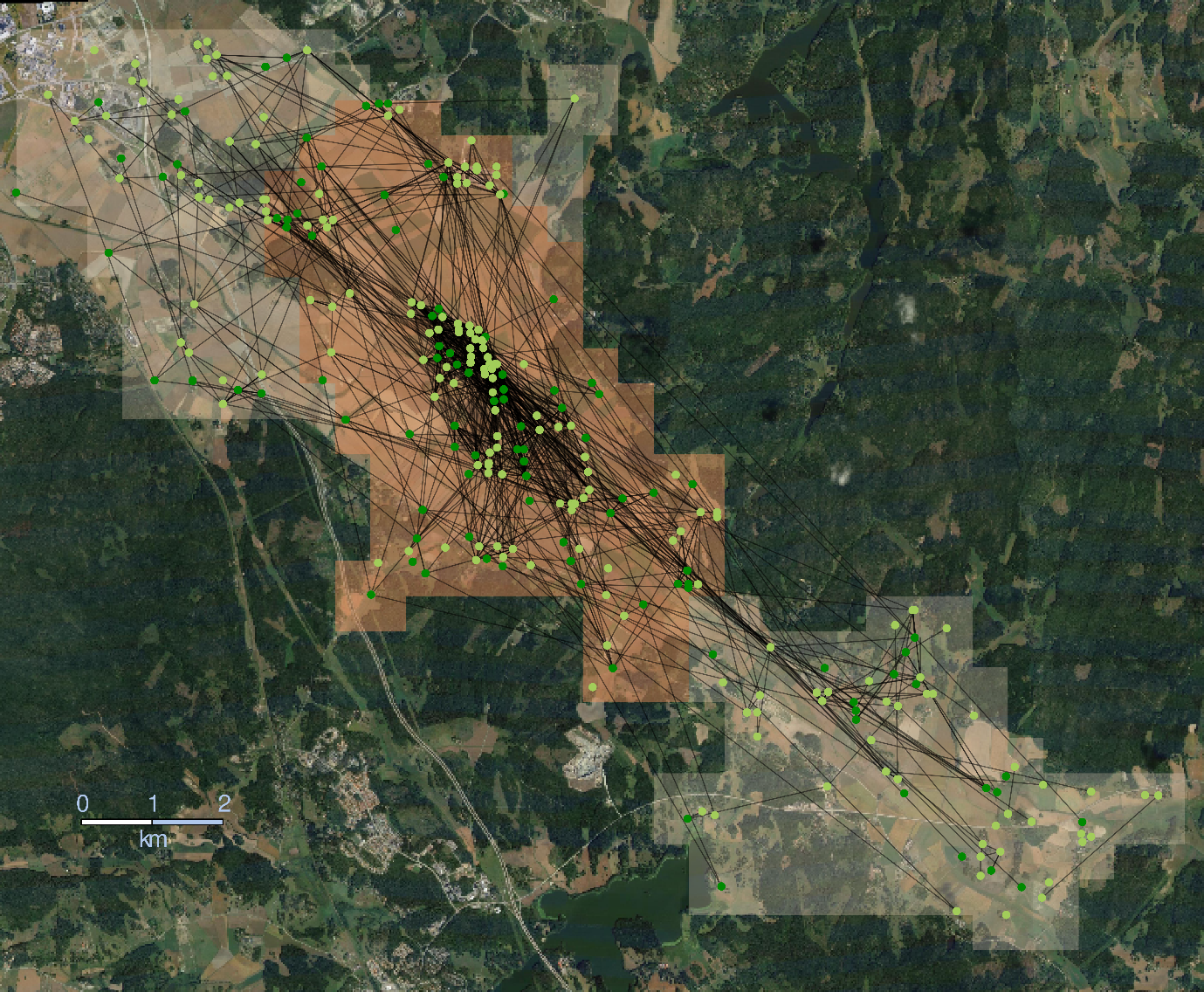


**Figure A3 1**: Study area near Uppsala, Sweden, and the locations of sites occupied by marked and resighted Northern Wheatears. Light green dots refer to sites with Short vegetation and dark green dots to sites with Tall vegetation (the last year it was assessed, since habitat type can vary from one year to the next). Observed between-year movements are connected with black lines. The orange and white transparent areas represent the delimitations of the central and periphery area, respectively, as used in the spatial CMR model component of the IPMs.

Estimated model parameters (see Appendix 1). **sigma.x** are the inter-annual location distances on the x and y axis (in km) for each age and habitat classes. Probstay_br_ correspond to the probability for a breeder to stay at the same site that is not predicted by the t distributions of mean 0 and variance **sigma.x** (that is, the probability of extra zero).

| **Parameter (95%CI)** | **Females** | **Males** |
| --- | --- | --- |
| sigma.x_fl,Short_ | 1.659 (1.453,1.890) | 2.030 (1.789,2.296) |
| sigma.x_fl,Tall_ | 2.264 (1.814,2.830) | 2.009 (1.669,2.408) |
| sigma.x_br,Short_ | 0.608 (0.551,0.673) | 0.450 (0.402,0.507) |
| sigma.x_br,Tall_ | 1.464 (1.259,1.695) | 0.831 (0.695,0.973) |
| Probstay_br,Short_ | 0.305 (0.262,0.350) | 0.469 (0.425,0.517) |
| Probstay_br, Tall_ | 0.178 (0.136,0.226) | 0.331 (0.277,0.389) |

Observed and estimated inter annual distances and probability to stay at the same breeding site. Estimated values are based on 10000 simulations from mean parameter estimates above and 95%CI for natal dispersal distances. Values here differ from values above because i) mean distances values result from movements on both the x and the y-axis and include individuals that do not move at all, ii) probabilities to stay result from both the t distributions with variance sigma.x and the t distribution with narrow-variance modelling the zero-inflated probability to move.

| **Parameter** | **Observed** | **Estimated** |
| --- | --- | --- |
| Mean natal dispersal distance of females from Short sites | 1.974 | 2.503 (2.217,3.467) |
| Mean natal dispersal distance of females from Tall sites | 2.304 | 3.467 (2.753,4.277) |
| Mean natal dispersal distance of males from Short sites | 2.246 | 3.092 (2.728,3.433) |
| Mean natal dispersal distance of males from Tall sites | 2.360 | 3.081 (2.524,3.612) |
| Mean distance of females breeding in Short sites | 0.602 | 0.642 |
| Mean distance of females breeding in Tall sites | 0.977 | 1.834 |
| Mean distance of males breeding in Short sites | 0.334 | 0.355 |
| Mean distance of males breeding in Tall sites | 0.407 | 0.839 |
| Probability to stay for females breeding in Short sites | 0.327 | 0.303 |
| Probability to stay for females breeding in Tall sites | 0.263 | 0.181 |
| Probability to stay for males breeding in Short sites | 0.492 | 0.479 |
| Probability to stay for males breeding in Tall sites | 0.409 | 0.330 |

Note that observed inter-annual distances are expected to be smaller than estimated distances as long distances are less likely to be observed due to the limited study area (Schaub & Royle, 2014). Note that for males the distribution of natal dispersal distances from Short and Tall habitat overlap nearly completely.


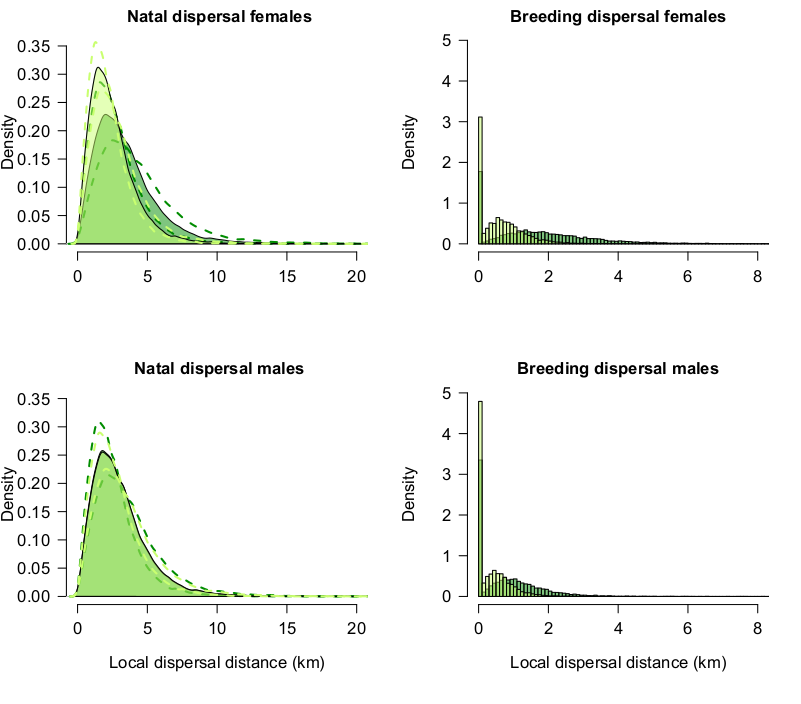


**Fig. A3 2** Estimated inter-annual distances. Based on 10000 simulations using mean estimated parameters, as well as their 95% CI for juveniles (dashed lines). Light green for Short habitat and dark green for Tall habitat. Note that for males the distribution of natal dispersal distances from Short and Tall habitat overlap nearly completely.


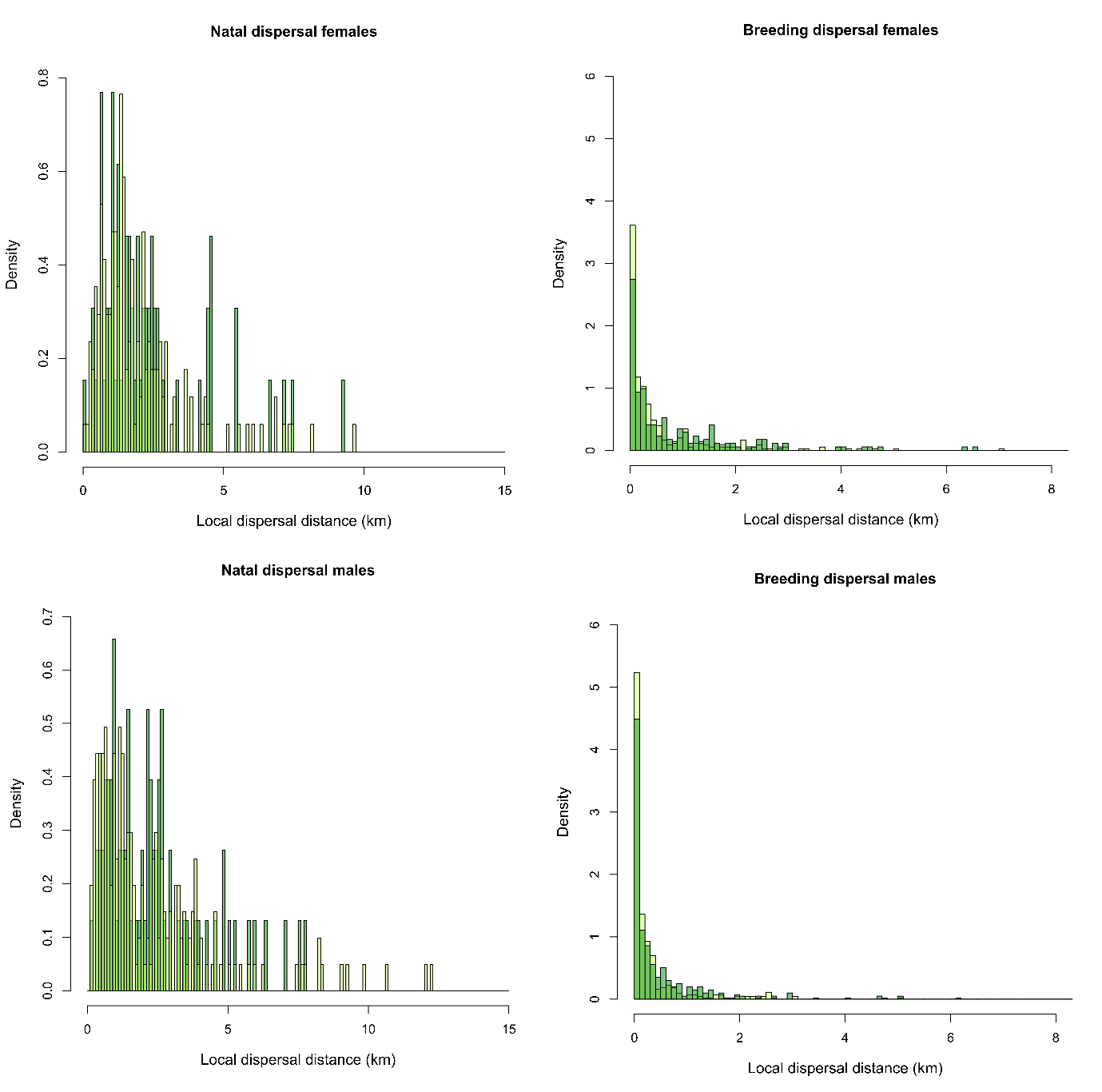


**Fig. A3 3** Observed inter-annual distances. Light green for Short habitat and dark green for Tall habitat.

MCMC iterations for some movement and re-sighting probability parameters mixed slowly (**Figures A3 3-3 6** below; different colours refer to the different MCMS chains). Although this is not ideal, we do not consider it as a major issue, notably because we were not primarily interested in estimating these parameters but in estimating survival, and estimates of first-year and breeder survival did satisfactorily converge (**Figures A3 7-3 8**). The mixing of chains for some movement and resighting probability parameters appeared to be a general problem and was much worse in a previous formulation of our model. In this previous version, we used a centered formulation of the location models (as in Schaub and Royle 2014). After we changed for a non-centered formulation (see **Appendix 1**) we achieved a much improved mixing (**Figures A3 3-3 6** below, see Papaspiliopoulos, Roberts & Skold (2007) for explanation on centered and non-centered formulations) although still not satisfactorily according to general norm (intermixing of different chains, low auto-correlation for each chain, R hat <1.1 while it varied from 1 to 1.27 for movement parameters). It is likely that we would have obtained more satisfactory mixing if we would have been able to increase the number of iterations substantially. However, this option was not realistic as our models already took 5 weeks to run. In the figures below the three colours (red, purple, green) represent the three chains based on 30000 iterations after an adaptation period of 29000, a burn-in of 1000 and thinning interval of 30.


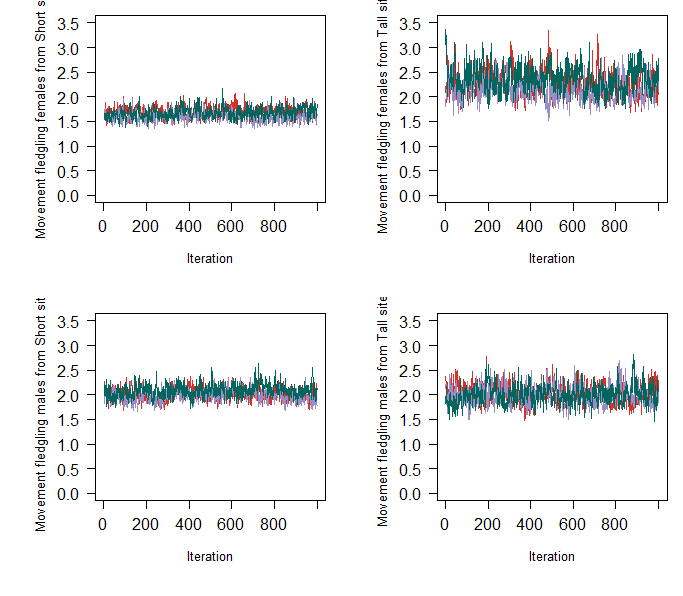


Figure A3 4


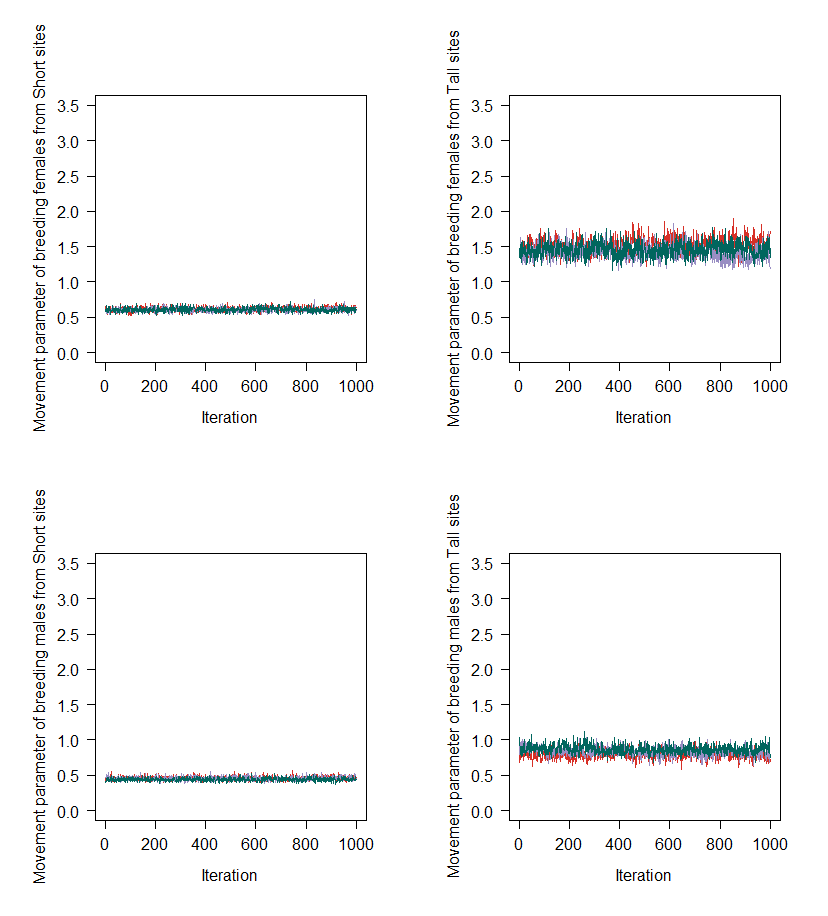


Figure A3 5


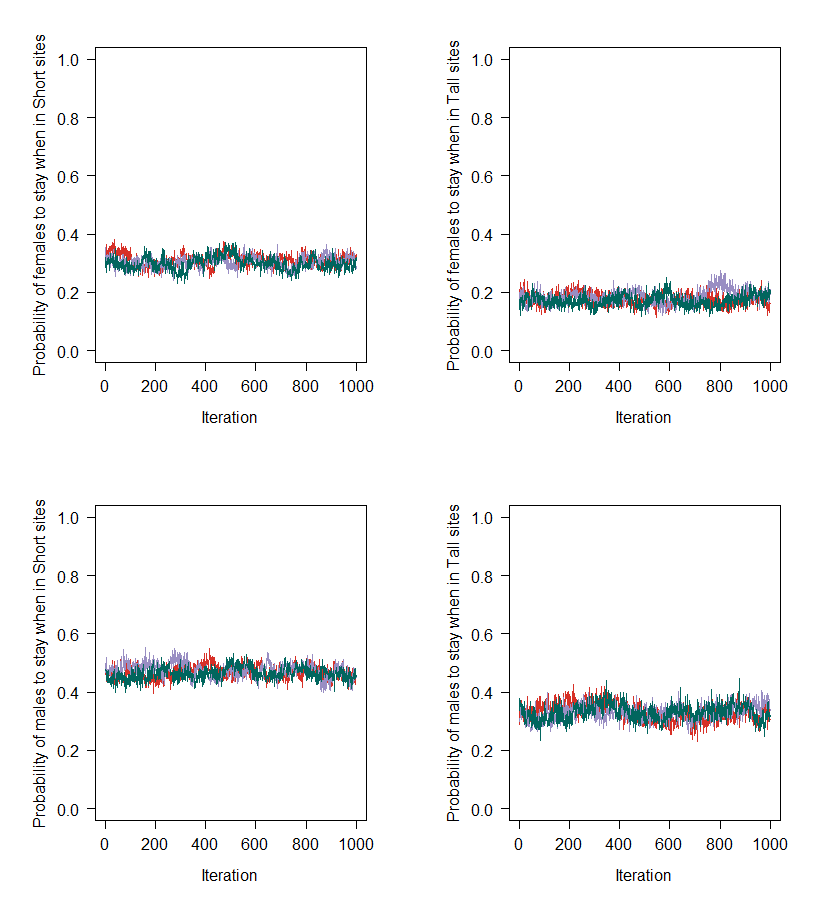
 *Figure A3 6*
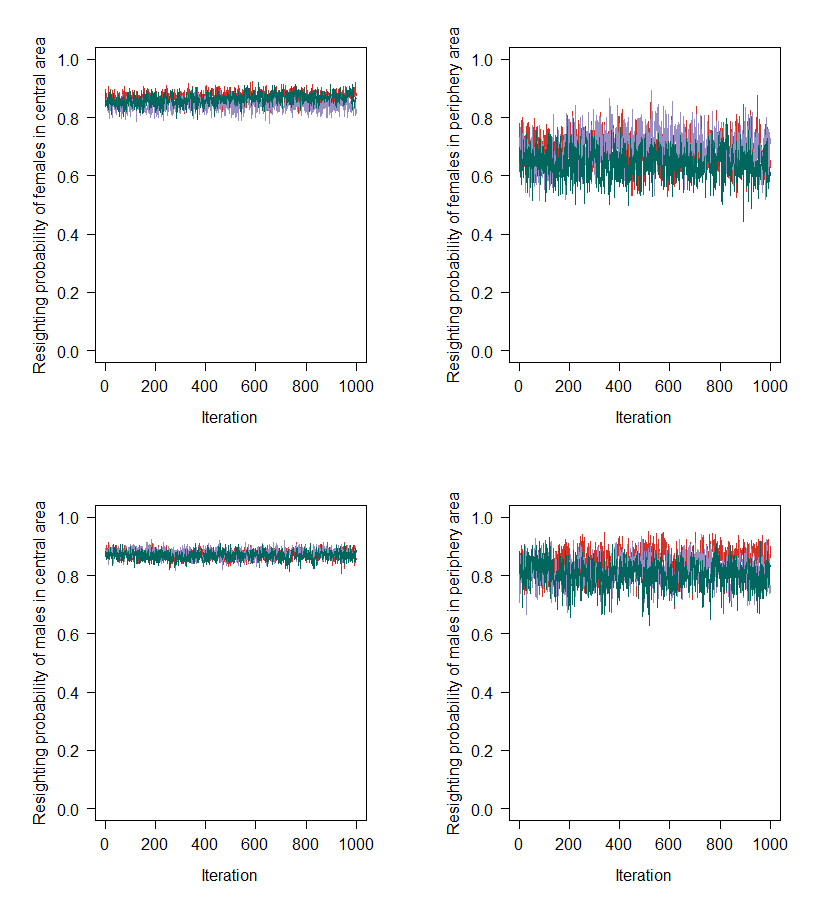


Figure A3 7


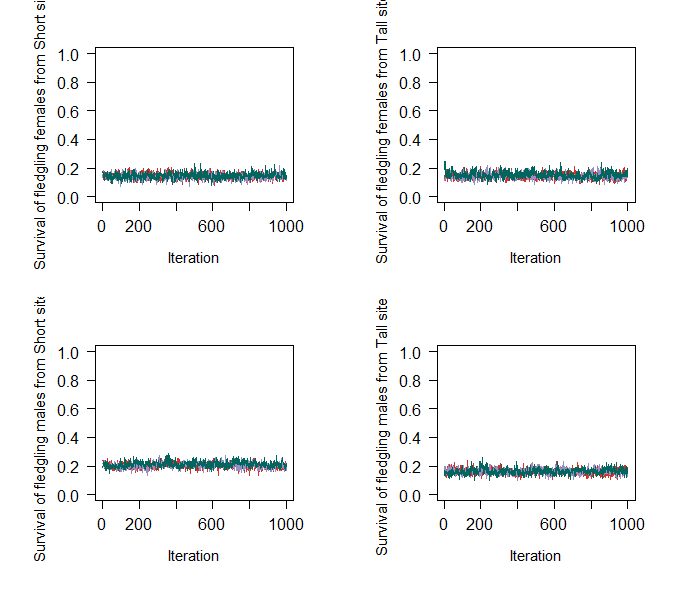
 Figure A3 8


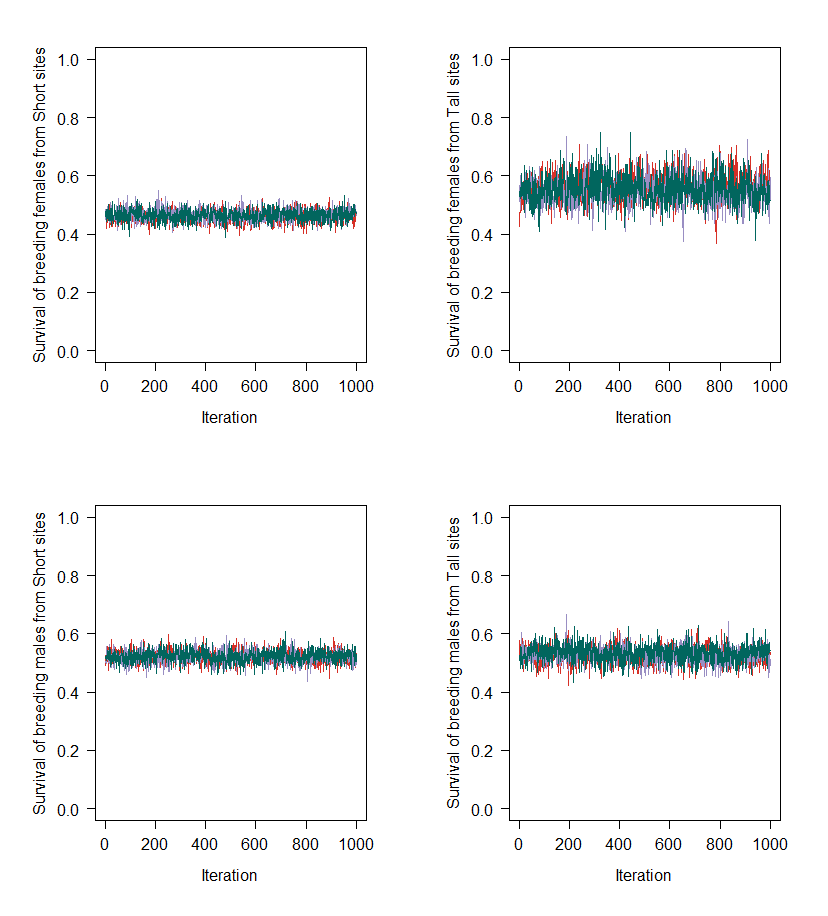


Figure A3 9

**Appendix 4: JAGS code to fit the spatially explicit integrated population model (including code for post predictive checking)**

**##### SCRIPT LEGEND**

**#description of the input data:**

#**N.TOT**: a 2*nyears matrix containing the observed number of occupied sites in Short (N.TOT[1,]) and Tall habitats (N.TOT[2,]) each year. Correspond to **C_Short_** and **C_Tall_** in the main text.

#**BS.sample.TOT**: a 2*nyears matrix containing the yearly number of monitored sites of each habitat for which the breeding success was known. **R_ht_** in the main text.

#**BS.success.TOT**: a 2*nyears matrix containing the number of successful sites (i.e. sites where successful reproduction was observed) counted each year for each habitat. **B_ht_** in the main text

#**fledg.sample.TOT**: 2*nyears matrix containing the yearly number of monitored successful sites of each habitat for which the number of fledglings was known. **S_ht_** in the main text.

#**rho.fledg**: 2*nyears matrix containing the number of fledglings from successful sites of each habitat each year MINUS “fledg.sample.TOT” **(**in order to use a Poisson distribution see explanation in main text). **F_ht_- S_ht_** in the main text.

#**nind.females**: numeric (3748) number of females in the capture resighting dataset. Subscript “**_i_”** in the main text

#**nyears**: numeric (24) number of years of study. Subscript “**_t_”** in the main text.

#**firstcap.females:** vector of length 3748 (nind.females) containing year of first capture for each female.

#**CMR.females:** nind.females*nyears matrix containing the observed event for each female each year. 1 = alive in Short habitats and 2= alive in Tall habitats, 3= alive but habitat uncertain, 4= not observed. **y_it_** in the main text.

#**latent.state.females:** nind.females*nyears matrix containing the latent state (when known) of each female each year. 1 = alive in Short habitats and 2= alive in Tall habitats. Note that it doesn’t contain any “3” as input data as we cannot be certain that an individual is dead. **z_it_** in the main text.

#**female.age**: nind.females*nyears matrix containing the age of each female. 1 if fledgling, 2 if older (breeder). 2 is entered for all years following the first known year (irrespective of whether the bird was resighted or not). Corresponds to the subscript “**_a_”** in the main text

#**females.x** and **females.y:** nind.females*nyears matrices containing the x and y coordinates of the sites where the birds were captured and resighted. **G_it_** in the main text.

#**ngrids.centre:** numeric (11) number of grids(rectangles) used to draw the central study area.

#**ngrids:** numeric (24) number of grids(rectangles) used to draw the totat study area.

#**grid.size.center.x** and **grid.size.center.y**: vectors of length 11 containing the length on the x and y axis of each grid composing the central area (in km)

#**grid.size.x and grid.size.y**: vectors of length 24 containing the length on the x and y axis of each grid composing the total study area (in km)

#**coord.central.x** and **coord.central.y**: vectors of length 11 containing the location on the x and y axis of the south west corner of each grid composing the central area (in km)

#**coord.studysite.x** and **coord.studysite.y**: vectors of length 24 containing the location on the x and y axis of the south west corner of each grid composing the total study area (in km)

**#Legend of the estimated parameters saved**

**#N**: array of dimension [2,nyears,samples,chains]. Number of occupied sites in Short (N[1,,,]) and Tall habitats (N[2,,,]) each year. Correspond to **NB_Short t_** and **NB_Tall t_** in the main text.

**#Nimm:** array of dimension [2,nyears,samples,chains]. Number of net immigrants in Short (Nimm[1,,,]) and Tall habitats (Nimm[2,,,]) each year. Correspond to **Imm_Short t_** and **Imm_Tall t_** in the main text.

**#BS:** array of dimension [2,nyears,samples,chains]. Breeding success probability in Short and Tall habitats each year. Correspond to **b_ht_** in the main text.

**#nfledg:** array of dimension [2,nyears,samples,chains]. Mean number of fledglings of the modelled sex per successful site in Short and Tall habitats each year. Correspond to **f_ht_** in the main text.

**#phipop.females:** array of dimension [2,2,nyears-1,samples,chains]. Survival rates of fledglings and breeders in Short and Tall habitats each year. Corresponds to the symbol **phi_aht_** in the main text.

**#psipop.females:** array of dimension [2,2,samples,chains]. Probability to change habitat between years for fledglings from and breeders in Short and Tall habitats. Corresponds to the symbol **psi_ah_** in the main text.

**#p.centre.females:** array of dimension [1,samples,chains]. Resighting probability of individuals in the central study area.

**#p.perif.females:** array of dimension [1,samples,chains]. Resighting probability of individuals in the periphery study area.

**#c.centre.females:** array of dimension [1,samples,chains]. Probability to know the state (habitat) of individuals in the central study area.

**#c.perif.females:** array of dimension [1,samples,chains]. Probability to know the state (habitat) of individuals in the periphery study area.

**#mov.coord.females** array of dimension [2,3,samples,chains] mean movements on the x or y axis of fledgling and breeders in Short and Tall habitats. Symbol **Sigma_move ah_** in main text.

**#prob.stay.females.old** array of dimension [3,samples,chains] mean probabitity of breeders in Short and Tall habitats to breed on the same territory the following year (extra zero movements, representing site fidelity). **Pstay_h_** in main text.

**#indexing legend:**

#i: individual ID number

#t: year

#h: habitat (Short=1, Tall=2)

#a: age (1= fledgling, 2= breeder)

#g: grid ID number. The central and the periphery study areas where subdivided into several rectangular areas (grids) in order to check whether individuals were inside or outside the central and the periphery areas.

model{

# Likelihood for count data (state-space model)

for (t in 1:nyears){

for(h in 1:2){

N.TOT[h,t]~dpois(N[h,t])

} #t

}#h

# System process [h,t]

for (t in 2:nyears){

Ntot[t]<-N[1,t]+N[2,t]

# Number of breeders in Short habitat

N[1,t]<-Npsi.rec[2,t-1]+(Nphi.rec[1,t-1]-Npsi.rec[1,t-1])+Npsipop[2,t-1]+(Nphipop[1,t-1]-Npsipop[1,t-1])+Nimm[1,t]

# Number of breeders in Tall habitat

N[2,t]<-Npsi.rec[1,t-1]+(Nphi.rec[2,t-1]-Npsi.rec[2,t-1])+Npsipop[1,t-1]+(Nphipop[2,t-1]-Npsipop[2,t-1])+Nimm[2,t]

#Number of net immigrants (can be negative)

Nimm[1,t]<-round(mean.Nimm[1,t])

Nimm[2,t]<-round(mean.Nimm[2,t])

}#t

###demographic stochasticity for the population level parameters

for (t in 2:nyears){

#number of breeders that survived and changed habitats (1 and 2 = habitat of origin: Short or Tall)

Npsipop[1,t-1]~ dbin(psipop.females[2,1],Nphipop[1,t-1])

Npsipop[2,t-1]~ dbin(psipop.females[2,2],Nphipop[2,t-1])

#number of breeders on each habitat that survived from year t-1 to t.

Nphipop[1,t-1]~ dbin(phipop.females[2,1,t-1],N[1,t-1])

Nphipop[2,t-1]~ dbin(phipop.females[2,2,t-1],N[2,t-1])

}#t

for (t in 2:nyears){

for(h in 1:2){

#number of surviving fledglings changing habitat

Npsi.rec[h,t-1]~ dbin(psipop.females[1,h],Nphi.rec[h,t-1])

#number of surviving fledglings

Nphi.rec[h,t-1]~ dbin(phipop.females[1,h,t-1],Nnfledg[h,t-1])

#number of fledglings of the modelled sex (here females) on each habitat, assuming sex ratio 1:1.

Nnfledg[h,t-1] ~ dbinom(0.5,NnfledgMF[h,t-1])

#number of fledglings of both sexes produced on each habitat

NnfledgMF[h,t-1]<-nfl[h,t-1]+NBS[h,t-1]

nfl[h,t-1]~dpois(NBS[h,t-1]*(2*nfledg[h,t-1]-1))

#number of succesful sites

NBS[h,t-1]~dbin(BS[h,t-1],N[h,t-1])

}#h

}#t

### Initial population sizes and priors

Ntot[1]<-N[1,1]+N[2,1]

nShort ~ dnorm(44, 0.001) I(1,200)

N[1,1] <- round(nShort)

nTall ~ dnorm(28, 0.001) I(1,200)

N[2,1] <- round(nTall)

#Number of immigrants at year 1 (this is just to give a value at time step 1 but these values are never used)

Nimm[1,1]<-1

Nimm[2,1]<-1

for(h in 1:2){

for(t in 1:(nyears-1)){

#yearly number of net immigrants

mean.Nimm[h,t+1] <- im[h,t]*N[h,t]

#net immigration rate

im[h,t] ~ dunif(-2,2)

} # time

} # site

#Likelihood for the number of fledglings from successful sites

#note that rho.fledg corresponds to the total number of fledglings from successful sites minus the total number of successful sites (in order to use a Poisson distribution)

for(u in 1:2){

for(t in 1:nyears){

rho.fledg[h,t]~dpois(fledg.sample.TOT[h,t]*(2*nfledg[h,t]-1))

}#t

}#h

for(h in 1:2){

for(t in 1:nyears){

#log link function

nfledg[h,t]<-exp(log.nfledg[h,t])

#mean and random temporal variation

log.nfledg[h,t]<-mu.nfledg[h]+epsilon.nfledg[h,t]

#random temporal variation

epsilon.nfledg[h,t] ~ dnorm(0,tau.nfledg[h])

#likelihood for Breeding success

BS.success.TOT[h,t] ~ dbin(BS[h,t],BS.sample.TOT[h,t])

#logit link function

BS[h,t]<-exp(logit.BS[h,t])/(1+exp(logit.BS[h,t]))

#mean and random temporal variation

logit.BS[h,t]<-mu.BS[h]+epsilon.BS[h,t]

#random temporal variation

epsilon.BS[h,t] ~ dnorm(0,tau.BS[h])

} # time

#Priors on random temporal variances of breeding success and number of fledglings (on logit and log scales respectively)

tau.BS[h]<-pow(sigma.BS[h],-2)

tau.nfledg[h]<-pow(sigma.nfledg[h],-2)

sigma2.BS[h]<-pow(sigma.BS[h],2)

sigma2.nfledg[h]<-pow(sigma.nfledg[h],2)

sigma.BS[h] ~ dt(0,1,1)T(0,)

sigma.nfledg[h] ~ dt(0,1,1)T(0,)

#Priors on mean breeding success and number of fledglings (on logit and log scales respectively

mu.BS[h] ~ dnorm(0,0.001)

mu.nfledg[h] ~ dnorm(0,0.001)

} # site

####data CMR

# Likelihood of capture-recapture data

for (i in 1:nind.females){

latent.state.females[i,firstcap.females[i]]<-CMR.females[i,firstcap.females[i]]

for (t in (firstcap.females[i]+1):nyears){

# Observation process

CMR.females[i,t] ~ dcat(po.females[latent.state.females[i,t],i,t-1,]+0.000001)

# State process

latent.state.females[i,t] ~ dcat(ps.females[latent.state.females[i,t-1],i,t-1,]+0.000001)

}#t

}#i

####### State-transition and observation matrices for the multistate model

for (i in 1:nind.females){

for (t in firstcap.females[i]:(nyears-1)){

#######define probabilities of capture plus state attribution

#[new state,recapt, indiv,time, event (CMR)]

po.females[1,i,t,1]<-p.females[i,t]*c.females[i,t]

po.females[1,i,t,2]<-0

po.females[1,i,t,3]<-p.females[i,t]*(1-c.females[i,t])

po.females[1,i,t,4]<-1-p.females[i,t]

po.females[2,i,t,1]<-0

po.females[2,i,t,2]<-p.females[i,t]*c.females[i,t]

po.females[2,i,t,3]<-p.females[i,t]*(1-c.females[i,t])

po.females[2,i,t,4]<-1-p.females[i,t]

po.females[3,i,t,1]<-0

po.females[3,i,t,2]<-0

po.females[3,i,t,3]<-0

po.females[3,i,t,4]<-1

#### Define probabilities of state S(t+1) given State(t)

#reminder: 1: Short habitats

# 2: Tall habitats

# 3:dead

#[initial state, indiv,t, new state]

ps.females[1,i,t,1]<-phi.females[i,1,t]*(1-psi.females[i,1,t])

ps.females[1,i,t,2]<-phi.females[i,1,t]*psi.females[i,1,t]

ps.females[1,i,t,3]<-1-phi.females[i,1,t]

ps.females[2,i,t,1]<-phi.females[i,2,t]*psi.females[i,2,t]

ps.females[2,i,t,2]<-phi.females[i,2,t]*(1-psi.females[i,2,t])

ps.females[2,i,t,3]<-1-phi.females[i,2,t]

ps.females[3,i,t,1]<-0

ps.females[3,i,t,2]<-0

ps.females[3,i,t,3]<-1

###survival and transitions age dependent (fledgling or breeder)

for(i in 1:nind.females){

for(h in 1:2){

for(t in firstcap.females[i]:(nyears-1)){

phi.females[i,h,t]<-phipop.females[female.age[i,t],u,t]

psi.females[i,h,t]<-psipop.females[female.age[i,t],u]

}#t

}#h

}#i

########### dispersal

#resighting probabilities and state certainties location dependent. Different between central area and periphery area and =0 if outside the study area.

p.females[i,t]<-r.centre.females[i,t+1]*p.centre.females+( r.females[i,t+1]- r.centre.females[i,t+1])*p.perif.females

c.females[i,t]<-r.centre.females[i,t+1]*c.centre.females+( r.females[i,t+1]- r.centre.females[i,t+1])*c.perif.females

}#t

}#i

p.centre.females~ dunif(0,1)

p.perif.females~ dunif(0,1)

c.centre.females~ dunif(0,1)

c.perif.females~ dunif(0,1)

###location on the x and y axes

for (i in 1:nind.females){

females.x.true[i,firstcap.females[i]]<-females.x[i,firstcap.females[i]]

females.y.true[i,firstcap.females[i]]<-females.y[i,firstcap.females[i]]

for (t in (firstcap.females[i]+1):nyears){

#this is just a trick in order to be able to use a non-centred formulation as we need a stochastic node for observed data.

females.x[i,t]~dnorm(females.x.true[i,t],100000)

females.y[i,t]~dnorm(females.y.true[i,t],100000)

#non-centred formulation of x and y locations

females.x.true[i,t]<-females.x.true[i,t-1]+eta.females.x.true[i,t]

females.y.true[i,t]<-females.y.true[i,t-1]+eta.females.y.true[i,t]

#location difference on the x and y axis between year t-1 to year t

eta.females.x.true[i,t]~ dt(0,mov.coord.x.females[i,t],5)

eta.females.y.true[i,t]~ dt(0,mov.coord.y.females[i,t],5)

#movements are (nearly) zero if individuals stay in their breeding territory (stay.females[i,t-1]=1), otherwise they depend on the age and habitat at time t-1.

mov.coord.x.females[i,t]<-stay.females[i,t-1]*100000+(1-stay.females[i,t-1])*tau.mov.females[female.age[i,t-1],latent.state.females[i,t-1]]

mov.coord.y.females[i,t]<-stay.females[i,t-1]*100000+(1-stay.females[i,t-1])*tau.mov.females[female.age[i,t-1],latent.state.females[i,t-1]]

#test whether an individual stay or not in their previous breeding site. Always 0 for fledglings (prob.stay.females set to zero for fledglings).

stay.females[i,t-1]~dbern(prob.stay.females[female.age[i,t-1],latent.state.females[i,t-1]])

# Test whether the actual location is in- or outside the state-space

for (g in 1:ngrids){

inside.females[i,t,g] <- step(females.x[i,t]-coord.studysite.x[g]) * step(coord.studysite.x[g]+grid.size.x[g]-females.x[i,t]) *

step(females.y[i,t]-coord.studysite.y[g]) * step(coord.studysite.y[g]+grid.size.y[g]-females.y[i,t])

} #g

r.females[i,t] <- max(inside.females[i,t,])

# Test whether the actual location is in- or outside the central area

for (g in 1:ngrids.centre){

inside.centre.females[i,t,g] <- step(females.x[i,t]-coord.central.x[g]) * step(coord.central.x[g]+grid.size.center.x[g]-females.x[i,t]) *

step(females.y[i,t]-coord.central.y[g]) * step(coord.central.y[g]+grid.size.center.y[g]-females.y[i,t])

} #g

r.centre.females[i,t] <- max(inside.centre.females[i,t,])

}#t

}#i

#note that here the indexing h=3 means dead state (so movement parameters cannot be identified for dead individuals, but they are estimated anyway)

for (h in 1:3){

for(a in 1:2){

# movement parameter

tau.mov.females[a,h] <-1/(mov.coord.females[a,h]^2)

mov.coord.females[a,h]~ dunif(0,3.5)

}#a

}#h

#probability to stay in the same site from a year to the next. This represent the extra zero movements due to site fidelity. Set to zero for fledglings.

for(h in 1:3){

prob.stay.females[1,h]<-0

prob.stay.females[2,h]<- prob.stay.females.old[h]

#zero inflation parameter for breeders

prob.stay.females.old[h]~dunif(0,1)

}#h

#random time variation on the logit scale for survival parameters

for(a in 1:2){

for(h in 1:2){

for(t in 1:(nyears-1)){

logit(phipop.females[a,h,t])<-eta.phi.females[a,h]+epsilon.phi.females[a,h,t]

epsilon.phi.females[a,h,t] ~ dnorm(0,tau.phi.females[a,h])

}#t

eta.phi.females[a,h] ~ dnorm(0,0.001) I(-3,3)

tau.phi.females[a,h]<-pow(sigma.phi.females[a,h],-2)

sigma.phi.females[a,h] ~ dt(0,1,1)T(0,)

#no temporal variation for the probability to change habitat type

psipop.females[a,h]~ dunif(0,1)

}#h

}#a

#POST PREDICTIVE CHECKS##

####evaluate fit for BS

for(h in 1:2){

for(t in 1:nyears){

eval.BS[h,t]<-BS[h,t]*BS.sample.TOT[h,t]

E.BS[h,t]<-pow((BS.success.TOT[h,t]-eval.BS[h,t]),2)/(eval.BS[h,t]*(1-BS[h,t])+0.01)

BS.success.new[h,t]~dbin(BS[h,t],BS.sample.TOT[h,t])

E.BS.new[h,t]<-pow((BS.success.new[h,t]-eval.BS[h,t]),2)/(eval.BS[h,t]*(1-BS[h,t])+0.01)

}#t

}#h

fit.BS<-sum(E.BS[,])

fit.BS.new<-sum(E.BS.new[,])

####evaluate fit for fledglings

for(h in 1:2){

for(t in 1:nyears){

eval.rho.fledg[h,t]<-fledg.sample.TOT[h,t]*(2*nfledg[h,t]-1)

E.rho.fledg[h,t]<-pow((rho.fledg[h,t]-eval.rho.fledg[h,t]),2)/(eval.rho.fledg[h,t]+0.01)

rho.fledg.new[h,t]~dpois(fledg.sample.TOT[h,t]*(2*nfledg[h,t]-1))

E.rho.fledg.new[h,t]<-pow((rho.fledg.new[h,t]-eval.rho.fledg[h,t]),2)/(eval.rho.fledg[h,t]+0.01)

}#t

}#h

fit.fledg<-sum(E.rho.fledg[,])

fit.fledg.new<-sum(E.rho.fledg.new[,])

####evaluate fit for Ncount

for(h in 1:2){

for(t in 1:nyears){

eval.N[h,t]<-N[h,t]

E.N[h,t]<-pow((N.TOT[h,t]-eval.N[h,t]),2)/(eval.N[h,t]+0.01)

N.TOT.new[h,t]~dpois(N[h,t])

E.N.new[h,t]<-pow((N.TOT.new[h,t]-eval.N[h,t]),2)/(eval.N[h,t]+0.01)

}#t

}#h

fit.N<-sum(E.N[,])

fit.N.new<-sum(E.N.new[,])

####evaluate fit CMR

CP.mean<-sum(CP.sum.ind)/sum(CP.sum.tot.ind)

CP.state.mean<-sum(CP.state.sum.ind)/sum(CP.sum.tot.ind)

for(i in 1:nind.females){

CP.sum.ind[i]<-sum(CP[i,(firstcap.females[i]+1):nyears])

CP.state.sum.ind[i]<-sum(CP.state[i,(firstcap.females[i]+1):nyears])

CP.sum.tot.ind[i]<-sum(CP.state.tot[i,(firstcap.females[i]+1):nyears])

}

for (i in 1:nind.females){

for(t in 1:nyears){

CP.state.tot[i,t]<-1

CP.tot[i,t]<-1

}

}

for (i in 1:nind.females){

for (t in (firstcap.females[i]+1):nyears){

CMR.new[i,t] ~ dcat(po.females[latent.state.females[i,t],i,t-1,]+0.000001)

CP[i,t] <- ifelse(CMR.new[i,t]==CMR.females[i,t],1,0)

latent.state.new[i,t] ~ dcat(ps.females[latent.state.females[i,t-1],i,t-1,]+0.000001)

CP.state[i,t] <- ifelse(latent.state.new[i,t]==latent.state.females[i,t],1,0)

}#t

}#i

############################ end of model fitting section

}

# Bundle data

data.females <- list( N.TOT=N.TOT,BS.sample.TOT=BS.sample.TOT,BS.success.TOT=BS.success.TOT,fledg.sample.TOT=fledg.sample.TOT,rho.fledg=rho.fledg,female.age=female.age,females.x=females.x,females.y=females.y,ngrids.centre=length(coord.center.grid[,1]),ngrids=length(coord.grid[,1]),grid.size.center.x=grid.size.center.x,grid.size.x=grid.size.x,grid.size.center.y=grid.size.center.y,grid.size.y=grid.size.y,coord.central.x=coord.central.x,coord.central.y=coord.central.y,coord.studysite.x=coord.studysite.x,coord.studysite.y=coord.studysite.y,nind.females = dim(CMR.females)[1],nyears=dim(CMR.males)[2], firstcap.females=firstcap.females,latent.state.females=latent.state.females,CMR.females=CMR.females)

###initial values

inits<-function(){list(

mov.coord.females=array(c(1,1,1,1,1,1),dim=c(2,3)),

prob.stay.females.old=c(0.3,0.3,0.3),

females.x=x.inits.females,

females.y=y.inits.females,

latent.state.females=ms.init.z.females(CMR.females,firstcap.females),

eta.phi.females=array(c(1/(1+exp(-0.1)),1/(1+exp(-0.1)),1/(1+exp(-0.52)), 1/(1+exp(-0.46))),dim=c(2,2)),

psipop.females=array(c(0.15,0.20,0.15,0.20),dim=c(2,2)),

p.centre.females=0.98,

p.perif.females=0.98,

c.centre.females=runif(1,0.6,0.99),

c.perif.females=runif(1,0.6,0.99))}

jm = jags.model(model, data=data.females, n.adapt=29000, inits=inits.females, n.chains=3)

burn.in=1000

update(jm, n.iter=burn.in)

samples=30000

n.thin=30

zj = jags.samples(jm, variable.names=c"fit.N","fit.N.new","fit.BS","fit.BS.new","fit.fledg","fit.fledg.new","CP.mean","CP.state.mean","N","Nimm","BS","nfledg","phipop.females","psipop.females","p.centre.females","p.perif.females","c.centre.females","c.perif.females","eta.phi.females","sigma.phi.females","mov.coord.females","prob.stay.females.old"),n.iter=samples, thin=n.thin)

**Appendix 5: Estimated emigration rates**

**Figure A5.1** Habitat-specific estimates (means and 95% credible intervals) of first-year and breeding emigration rates for females

**Figure A5. 2** Habitat-specific estimates (means and 95% credible intervals) of first-year and breeding emigration rates for males

**Figure A5. 3** Habitat-specific emigration rates of fledglings and breeding females across years (means and 95% credible intervals).

**Figure A5. 4** Habitat-specific emigration rates of fledglings and breeding males across years (means and 95% credible intervals).

Estimates of emigration rates were derived based on the spatial-CMR component of the IPM as the number of marked individuals of a given age and in a given habitat inside the study area in year t that survived and moved outside the study area at year t+1 divided by the number of individuals of this given age and habitat inside the study area in year t that survived at year t+1 (and could be either in or outside the study area).

Estimates were derived from the female and male IPMs, based on 21000 samples of 2 chains (17000 for the chains of the male model) thinned every 20^th^ iteration after an adaptation phase of 9000 iterations and a burn in of 5000 iterations (9000 for the male model).

**#SCRIPT TO CALCULATE THE AGE AND HABITAT SPECIFIC IMMIGRATION RATES IN R USING POSTERIOR SAMPLES FROM JAGS**

**# n.ind: number of individuals**

**# noyears: number of years minus one**

**# samples: number of iterations saved**

**#chains: number of chains**

**# r.males[n.ind,noyears+1,samples,chains] tells if an individual is inside (1) or outside (0) the study area**

**#latent.state.male[n.ind,noyears+1,samples,chains] are the state of the individuals: 1= alive in short habitat, 2= alive in Tall habitat, 3=dead**

**#male.age[n.ind,noyears+1] is the age class. 1=fledgling and 2= breeder**

**#Here we state that the individuals were inside the study area at first capture**

for(i in 1:length(r.males[,1,1,1])){

r.males[i,firstcap.males[i],,]<-1

}**#i**

**#function to calculate the emigration rate for each age class and habitat**

**emigr.function<-**function(age,state_habitat,inside_area,firstcap,n.ind,noyears,samples,chains){

alive<-array(0,dim=c(2,2,n.ind,noyears,samples,chains))

alive.emigr<-array(0,dim=c(2,2,n.ind,noyears,samples,chains))

**#loop over individuals**

for(i in 1:n.ind){

**#loop over years (from year of first capture)**

for(t in firstcap[i]:noyears){

**#loop over age**

for(a in 1:2){

**#loop over habitats (1 for Short and 2 for Tall)**

for(h in 1:2){

**#check if the individual is of age a in habitat h and inside the study area at time t and survived to time t+1 (state_habitat<3)**

if(age[i,t]==a)alive[a,h,i,t,,]=(state_habitat[i,t,,]==h) & (inside_area[i,t,,]) & (state_habitat[i,t+1,,]<3)

**#in addition, check if the individual was outside the study are at time t+1 (if it emigrated or not)**

alive.emigr[a,h,i,t,,] = alive[a,h,i,t,,] & !inside_area[i,t+1,,]

**}#habitats**

**}#age**

**}#year**

**}#individuals**

emigr.rate<-array(0,dim=c(2,2,noyears,samples,chains))

**###here we calculate the emigration rate as the number of individuals of each age and habitat inside the study area that survive and emigrate, divided by the number of this age and habitat inside the study area that survived, no matter where**

emigr.rate<-apply(alive.emigr, c(1:2,4:6), sum, na.rm = TRUE)/apply(alive , c(1:2,4:6), sum, na.rm = TRUE)

return(emigr.rate)

}

emigr.rate<-emigr.function(male.age,latent.state.males,r.males,firstcap.males,n.ind,noyears,samples,chains)

**#legend of the indexing for emigration rate: emigr.rate[age,habitat,year,sample,chain]**

**#emigration rate for fledglings from Short habitats**

emigr.rate.fled.short<- emigr.rate[1,1,,,]

**#emigration rate for fledglings from Tall habitats**

emigr.rate.fled.tall<- emigr.rate[1,2,,,]

**#emigration rate for breeders in Short habitats**

emigr.rate.br.short<- emigr.rate[2,1,,,]

**#emigration rate for breeders in Tall habitats**

emigr.rate.br.tall<- emigr.rate[2,2,,,]

**Appendix references**

Acker, P. (2017). *Life-history decisions of larids in spatio-temporally varying habitats: where and when to breed* (PhD Thesis). Université de Toulouse, Université Toulouse III-Paul Sabatier.

Gelman, A., Meng, X.-L., & Stern, H. (1996). Posterior predictive assessment of model fitness via realized discrepancies. *Statistica Sinica*, 733–760.

Gimenez, O., Lebreton, J.-D., Choquet, R., & Pradel, R. (2018). R2ucare: An r package to perform goodness-of-fit tests for capture–recapture models. *Methods in Ecology and Evolution*.

Greenhill, B., Ward, M. D., & Sacks, A. (2011). The separation plot: A new visual method for evaluating the fit of binary models. *American Journal of Political Science*, *55*(4), 991–1002.

Papaspiliopoulos, O., Roberts, G. O., & Sköld, M. (2007). A general framework for the parametrization of hierarchical models. *Statistical Science*, 59–73.

Paquet, M., Arlt, D., Knape, J., Low, M., Forslund, P., & Pärt, T. (2019). Quantifying the links between land use and population growth rate in a declining farmland bird. *Ecology and Evolution*, *9*(2), 868–879. doi: 10.1002/ece3.4766

Schaub, M., & Royle, J. A. (2014). Estimating true instead of apparent survival using spatial Cormack–Jolly–Seber models. *Methods in Ecology and Evolution*, *5*(12), 1316–1326.

**Table A1:** An example to illustrate the estimation of habitat-specific net immigration.

|  | total | inside local population: habitat A | outside local population |
| --- | --- | --- | --- |
| number of breeders from count data in habitat A |  | 15 |  |
| projected number of breeders in habitat A that originate from the local population in the previous year (either habitat A or B) | 10 | X1 | X2 |
| **Net immigration rate** |  | 15-10=5 |  |

From the population count data we get the estimate of 15 individuals breeding inside the local population. From breeding and resighting data we estimate that there should be a total of 10 individuals based on what has been breeding in the previous year. We do not know where those individuals end up, i.e. we do not know how many of them inside or outside the local population. But we can see that while we estimate there should be 10 breeders there are 15, hence 5 are inferred to be immigrants.

**Table A2:**

| **Parameter** | **Temporal variance (95% BCI)** |
| --- | --- |
| **N_Short_** | 136.9 (81.8-204.9) |
| **N_Tall_** | 104.4 (66.6-152.9) |
| **b_Short_** | 3.31×10^-3^(9.96×10^-4^-6.24×10^-3^) |
| **b_Tall_** | 2.21×10^-3^(1.52×10^-5^-6.03×10^-3^) |
| **f_Short_** | 4.51×10^-3^(8.07×10^-6^-2.08×10^-2^) |
| **f_Tall_** | 2.57×10^-2^(1.20×10^-4^-7.72×10^-2^) |
| **φ*_fl_* _Short F_** | 5.42×10^-3^(2.23×10^-3^-9.51×10^-3^) |
| **φ*_fl_* _Tall F_** | 1.11×10^-3^(1.30×10^-6^-4.99×10^-3^) |
| **φ*_fl_* _Short M_** | 2.50×10^-3^ (3.72×10^-5^-6.72×10^-3^) |
| **φ*_fl_* _Tall M_** | 7.54×10^-4^ (5.10×10^-7^-3.76×10^-3^) |
| **φ*_br_* _Short F_** | 1.56×10^-3^ (5.64×10^-6^-6.41×10^-3^) |
| **φ*_br_* _Tall F_** | 1.88×10^-2^ (1.19×10^-3^-4.26×10^-2^) |
| **φ*_br_* _Short M_** | 2.58×10^-3^ (4.97×10^-6^-8.73×10^-3^) |
| **φ*_br_* _Tall M_** | 2.32×10^-3^ (3.08×10^-6^-1.04×10^-2^) |
| **Ω _Short F_** | 0.10 (0.05 -0.20) |
| **Ω _Tall F_** | 0.17 (0.06 -0.38) |
| **Ω _Short M_** | 0.11 (0.05 -0.21) |
| **Ω _Tall M_** | 0.25 (0.09 -0.58) |

Estimated coefficients of temporal variance of the demographic parameters for females (**F**) and males (**M**) with associated 95% Bayesian credible intervals (BCI) from the spatially explicit IPM. **N**: the number of breeding pairs in each habitat type, **b**: breeding success, **f**: number of fledglings at successful sites, **φ**: breeder (*br*) and first-year (*fl*) survival probabilities, Ω: net immigration rate, **Short** and **Tall**: sites with Short or Tall ground vegetation. All parameters were estimated from the spatially explicit integrated population models, i.e. accounting for emigration as estimated from observed local movements.

| **Parameter** | **Mean (95% Bayesian Credibility Intervals)** |
| --- | --- |
| **ψ*_fl_* _Short F_** | 0.412 (0.343,0.484) |
| **ψ*_fl_* _Tall F_** | 0.573 (0.458,0.689) |
| **ψ*_fl_* _Short M_** | 0.482 (0.415,0.550) |
| **ψ*_fl_* _Tall M_** | 0.561 (0.447,0.670) |
| **ψ*_br_*_Short F_** | 0.225 (0.185,0.266) |
| **ψ*_br_*_Tall F_** | 0.546 (0.473,0.614) |
| **ψ*_br_* _Short M_** | 0.212 (0.175,0.251) |
| **ψ*_br_* _Tall M_** | 0.514 (0.448,0.578) |
| **p_centre F_** | 0.860 (0.813,0.901) |
| **p_perif F_** | 0.675 (0.554,0.798) |
| **p_centre M_** | 0.873 (/0.841,0.903) |
| **p_periph M_** | 0.823 (0.722,0.914) |
| **c_centre F_** | 0.982 (0.972,0.991) |
| **c_periph F_** | 0.924 (0.873,0.961) |
| **c_centre M_** | 0.967 (0.953,0.978) |
| **c_periph M_** | 0.901 (0.857,0.938) |

**Table A3:** estimates of transition probabilities **ψ**, re/sighting probabilities **p** and state certainties **c**. **fl**: transition probabilities for juveniles (between natal and first-breeding site) and ***br*** for breeders (between consecutive breeding sites), **Short** and **Tall**: the two habitat types, **F** and **M:** females and males, and **centre** and **periph**: the central and peripheral study areas.
